# The diversity of ice algal communities on the Greenland Ice Sheet as revealed by oligotyping

**DOI:** 10.1099/mgen.0.000159

**Published:** 2018-03-16

**Authors:** Stefanie Lutz, Jenine McCutcheon, James B. McQuaid, Liane G. Benning

**Affiliations:** ^1^​ GFZ German Research Centre for Geosciences, Telegrafenberg, 14473 Potsdam, Germany; ^2^​ School of Earth and Environment, University of Leeds, Leeds LS2 9JT, UK

**Keywords:** Ice algae, Greenland Ice Sheet, albedo, oligotyping, Illumina, OTU

## Abstract

The Arctic is being disproportionally affected by climate change compared with other geographic locations, and is currently experiencing unprecedented melt rates. The Greenland Ice Sheet (GrIS) can be regarded as the largest supraglacial ecosystem on Earth, and ice algae are the dominant primary producers on bare ice surfaces throughout the course of a melt season. Ice-algal-derived pigments cause a darkening of the ice surface, which in turn decreases albedo and increases melt rates. The important role of ice algae in changing melt rates has only recently been recognized, and we currently know little about their community compositions and functions. Here, we present the first analysis of ice algal communities across a 100 km transect on the GrIS by high-throughput sequencing and subsequent oligotyping of the most abundant taxa. Our data reveal an extremely low algal diversity with *Ancylonema nordenskiöldii* and a *Mesotaenium* species being by far the dominant taxa at all sites. We employed an oligotyping approach and revealed a hidden diversity not detectable by conventional clustering of operational taxonomic units and taxonomic classification. Oligotypes of the dominant taxa exhibit a site-specific distribution, which may be linked to differences in temperatures and subsequently the extent of the melting. Our results help to better understand the distribution patterns of ice algal communities that play a crucial role in the GrIS ecosystem.

## Data Summary

DNA sequences have been deposited to the European Nucleotide Archive (ENA) under accession number ERP104425 and can be accessed under https://figshare.com/s/2c10e295e7b6f20bb16a.

**Impact Statement**

Pigmented algae are ubiquitous and the dominant primary producers on the Greenland Ice Sheet (GrIS), which is the largest supraglacial ecosystem on Earth. Their role and importance in changing melt rates has only recently been recognized, and we know very little about the algal distribution patterns. Here, we not only present the first high-throughput sequencing analysis of these algal communities, but also show that a conventional operational taxonomic unit (OTU) clustering approach does not sufficiently reveal their diversity. Only an oligotyping approach revealed the site-specific distribution of the species, which may be linked to differences in temperatures and hence the extent of the melting. Our results will help to better understand the distribution patterns of these algal communities that play a crucial role in the GrIS ecosystem, and thus are of interest for the cryosphere and climate community. In addition, the appropriate choice of a similarity threshold to bin sequences into OTUs and delineate species is currently a highly debated issue. Thus, the results of the oligotyping approach presented are informative for a wide audience.

## Introduction

The Greenland Ice Sheet (GrIS) is an important component of Earth’s cryosphere; it plays a crucial role as a freshwater reservoir and is an integral component of the Earth system processes driving current sea level rise and climate change [1, 2]. Due to the extensive area over which surface melting occurs on the GrIS, which can be up to 100 % during extreme melt events [3], the GrIS can be regarded as the largest supraglacial ecosystem on Earth. The discovery of ice algal communities on the GrIS dates back to the polar explorers Adolf Erik Nordenskiöld and Sven Berggren during their expeditions in the 1870s [4, 5]. However, only in the last decade have we recognized their importance with regard to primary production and decrease in surface albedo [6–9].

After the onset of melting and the disappearance of the snow cover, ice algae are the dominant primary producers on bare ice surfaces [6, 10]. The most prominent taxa are members of the Zygnematales, comprising *Ancylonema nordenskiöldii* Berggren 1871, *Mesotaenium berggrenii* Lagerheim 1892 and *Cylindrocystis brebissonii f*. *cryophila* Kol 1942 [11–13]. Ice algae produce a brown vascular pigment with a tannin structure identified as purpurogallin carboxylic acid-6-*O*-β-d-glucopyranoside as a means of protecting their photosystematic apparatus from photoinhibition [14]. Together with airborne-delivered impurities such as mineral dust, black carbon and bacteria, this algal-derived pigment causes a darkening of the ice surface, which in turn decreases albedo and increases melt rates [6, 10, 15–19]. The availability of liquid water is restricted to the short summer melt season, resulting in ice algae having to cope with desiccation stress for most of the year. In contrast to snow algae [20, 21], ice algae lack a flagellated stage and therefore cannot actively move upwards from the bottom ice into the melting snow layer at the onset of the melt season. Ice algae are restricted to the ice surface, where they overwinter in a frozen state [12]. *A. nordenskiöldii* has so far only been described in polar settings [6, 8], whereas *M. berggrenii* and *Cylindrocystis brebissonii* also occur in alpine settings [7, 11, 12]. The evaluation of the ice algal community composition on the GrIS has, to this point, been based only on microscopy and morphological descriptions [6, 22, 23]. In contrast, high-throughput sequencing allows for a more comprehensive assessment of the microbial community composition of an environment. In Arctic settings, such an approach has, thus far, targeted only snow algal communities [24–27], or prokaryotic communities on ice surfaces or in cryoconite holes [28–30]. The lack of application of this technique to resolve the eukaryotic assemblage on the GrIS is remarkable, particularly because these ice algal communities are important contributors to both primary production and albedo reduction [6].

While there is a consensus that the 16S rRNA gene is the most suitable marker for prokaryotic communities, there is no clear choice for a eukaryotic counterpart. ITS2 has been suggested as a suitable barcode for plants and fungi due to its high taxonomic resolution [31, 32]. However, ITS2 likely has limited use in environmental barcoding for ice algal communities due to the lack of reference sequences and universal priming [33], as well as due to its high intragenomic variation [34, 35]. Whilst the 18S rRNA gene is known to have more limited specificity, the availability of universal primers and comprehensive reference databases confer a higher universal applicability. Hence, a two-gene approach is advisable [36].

The nature of high-throughput sequencing to produce large datasets prompts the necessity of clustering sequences into operational taxonomic units (OTUs) at an apparently arbitrary similarity threshold (mostly 97 %). It is not clear whether strains assigned to one OTU actually represent a single species with similar eco-physiological functions [37]. Furthermore, these OTUs can be rather heterogeneous, and subtle differences of 1 bp in a gene can represent considerable genomic or ecological variation [38], and would be even overlooked at a stringent 99 % similarity threshold. Therefore, we used oligotyping, a high-resolution method that uses Shannon entropy to evaluate the most information-rich nucleotide position in an amplicon data set [39, 40]. Shannon entropy has a scalable capacity to quantify the uncertainty among the nucleotide columns of the alignment. The entropy of each position [on a scale from 0 (none) to 1 (highest entropy)] in the alignment is evaluated, and nucleotide positions with the highest entropy values are used for oligotyping. Amplicon sequencing comes with several sources of bias and distinct error patterns [41]. However, most sequencing errors are to a certain extent distributed randomly along the alignments and are more frequent towards the end of the reads [39]. On the basis of empirical observations, Eren *et al.* [39] found that, in general, the Illumina platforms generate sequencing errors with entropy values around and below 0.2. Therefore, the Shannon entropy approach allows the differentiation between true genomic variation and sequencing noise, and can segregate sequences that differ by as little as a single nucleotide. Thus, it is a powerful tool to reveal previously overlooked distribution patterns for microbial communities.

Here, we present the first analysis of ice algal communities on the GrIS Sheet by high-throughput sequencing combined with subsequent oligotyping of the most abundant taxa. We hypothesize that similar to snow algal communities [24–27] the overall species diversity in ice algae is low, but oligotyping has the potential to unveil hidden patterns in community composition. This will help us to better understand these algal communities that play a crucial role in the GrIS environment.

## Methods

### Field sites

**Fig. 1.**
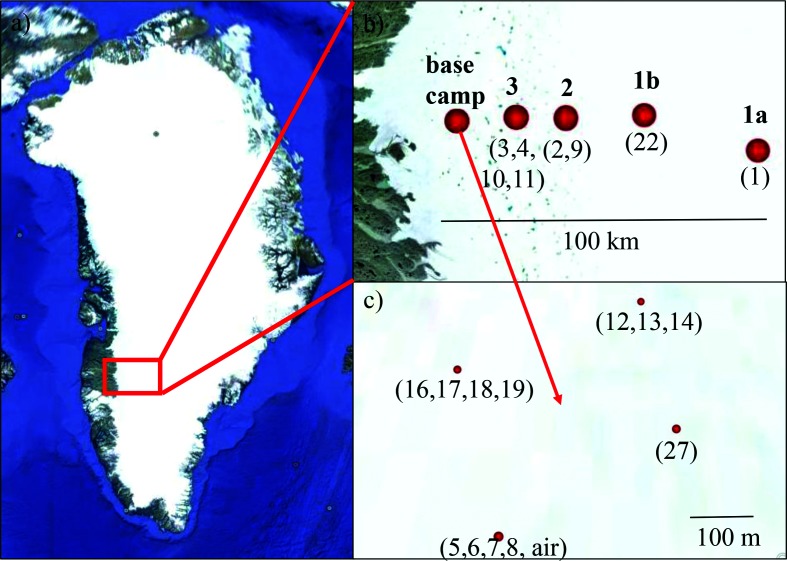
Map showing the location of field sites in southwest Greenland (a) across a 100 km transect from the base camp to further inland (b). Sites 1a, 2 and 3 were sampled on 27 July and sites 1b, 2 and 3 were sampled on 5 August 2016. The samples around the base camp were collected over a 3-week period (c). Labels in bold type above the red dots represent site names and those below in parentheses indicate sample numbers. Full details can be found in Table 1. (Image source: Google Earth, 23.06.2017).

Samples were collected on the GrIS between 27 July and 14 August 2016 (Table 1). All field sites were in the so called ‘dark zone’, which is situated in the western ablation zone of the GrIS and characterized by high particulate loading [42]. The field sites were spread along a 100-km-long transect. Field sites 1a and 1b still had a substantial snow cover at the time of sampling, whereas sites 2 and 3 and the base camp (S6) were snow-free (Fig. 1, see Table 1 for GPS coordinates). The sites not at the base camp (sites 1 to 3) were sampled once at the very beginning (1a), and once at the very end (1b) of the sampling campaign. Note that due to logistical reasons site 1a was at a slightly different location from site 1b. A total of 21 samples were collected and analysed. These comprised 11 dirty ice samples (macroscopically visible particles), two clean snow and two clean ice samples (without macroscopically visible particles), two dispersed cryoconite samples (larger and darker particles than dirty ice), two dirty snow samples (remnant small snow patches) and one air sample. Two sites within the base camp were sampled twice within one day to assess the reproducibility (GrIS_5/7 and GrIS_6/8). In addition, these two sites were in the close vicinity (~5 m) of each other to enable evaluation of the local spatial heterogeneity.

**Table 1.** Overview of all samples collected in SW Greenland for ice algal community characterization

**Sample label**	**Sample type**	**Site**	**Date of collection**	**GPS location**	**Elev. (m)**
GrIS16_1	Clean snow	1a	27/07/2016	N 67º 00.036′ W 47º 01.545′	1854
GrIS16_2	Dirty ice	2	27/07/2016	N 67º 05.717′ W 48º 30.648′	1385
GrIS16_3	Dirty ice	3	27/07/2016	N 67º 05.392′ W 48º 53.708′	1238
GrIS16_4	Dirty snow	3	27/07/2016	N 67º 05.477′ W 48º 53.573′	1236
GrIS16_12	Clean ice	Base camp	30/07/2016	N 67º 04.719′ W 49º 21.025′	1023
GrIS16_13	Dispersed cryoconite	Base camp	30/07/2016	N 67º 04.708′ W 49º 21.022′	1023
GrIS16_14	Dirty ice	Base camp	30/07/2016	N 67º 04.697′ W 49º 21.010′	1018
GrIS16_air	Air	Base camp	31/07/2016	N 67º 03.597′ W 49º 22.599′	1011
GrIS16_5	Dirty ice	Base camp	31/07/2016	N 67º 04.462′ W 49º 21.254′	1015
GrIS16_6	Dirty ice	Base camp	31/07/2016	N 67º 04.470′ W 49º 21.285′	1013
GrIS16_7	Dirty ice	Base camp	01/08/2016	N 67º 04.462′ W 49º 21.254′	1015
GrIS16_8	Dirty ice	Base camp	01/08/2016	N 67º 04.470′ W 49º 21.285′	1013
GrIS16_16	Clean ice	Base camp	02/08/2016	N 67º 04.616′ W 49º 21.427′	1013
GrIS16_17	Dirty ice	Base camp	02/08/2016	N 67º 04.616′ W 49º 21.427′	1014
GrIS16_18	Dispersed cryoconite	Base camp	02/08/2016	N 67º 04.626′ W 49º 21.368′	1011
GrIS16_19	Dirty ice	Base camp	02/08/2016	N 67º 04.602′ W 49º 21.428′	1013
GrIS16_22	Clean snow	1b	05/08/2016	N 67º 06.310′ W 47º 54.335′	1578
GrIS16_9	Dirty ice	2	05/08/2016	N 67º 05.389′ W 48º 30.657′	1402
GrIS16_10	Dirty ice	3	05/08/2016	N 67º 05.487′ W 48º 53.984′	1236
GrIS16_11	Dirty snow	3	05/08/2016	N 67º 05.524′ W 48º 53.863′	1237
GrIS16_27	Clean ice	Base camp	14/08/2016	N 67º 04.558′ W 49º 20.947′	1025

### Field sampling

Snow and ice samples were collected into sterile 50 ml centrifuge tubes or sterile sampling bags and thawed slowly on-site at ambient temperatures (~5–10 °C). Samples with high biomass were concentrated by gravimetric settling of the particles and stored in 5 ml cryo-tubes. Clean snow and clean ice samples were filtered (5 l of melted snow/ice water) through sterile Nalgene single-use filtration units (pore size 0.22 µm). The air sample was collected for 8 h into sterile milli-Q water at a rate of 300 l min^−1^ using a Coriolis air sampler (Bertin Instruments) and filtered as described above. All filtered samples were immediately transferred and transported back to the lab in a cryo-shipper cooled to liquid nitrogen temperature.

### DNA extractions and sequencing

DNA stored on filters (clean snow, clean ice, air) was extracted using the PowerWater DNA Isolation kit (MoBio Laboratories). All other higher biomass samples were processed using the PowerSoil DNA Isolation kit (MoBio Laboratories). The 18S rRNA and ITS2 amplicons were prepared according to the Illumina ‘16S Metagenomic Sequencing Library Preparation’ guide (https://support.illumina.com/content/dam/illumina-support/documents/documentation/chemistry_documentation/16s/16s-metagenomic-library-prep-guide-15044223-b.pdf). In brief, 18S rRNA genes were amplified using the eukaryotic primers 528F (5′ GCGGTAATTCCAGCTCCAA) and 706R (5′ AATCCRAGAATTTCACCTCT; [43]) spanning the V4–V5 hypervariable regions. ITS2 genes were amplified using the primers 5.8SbF (5′ GATGAAGAACGCAGCG; [44]) and ITS4R (5′ TCCTCCGCTTATTGATATGC; [45]). All primers were tagged with the Illumina adapter sequences. PCRs were performed using KAPA HiFi HotStart ReadyMix. Initial denaturation at 95 °C for 3 min was followed by 25 cycles of denaturation at 95 °C for 30 s, annealing at 55 °C for 30 s and elongation at 72 °C for 30 s. Final elongation was at 72 °C for 5 min. All PCRs were carried out in reaction volumes of 25 µl. All pre-amplification steps were done in a laminar flow hood with certified DNA-free plasticware and filter tips. Amplicons were barcoded using the Nextera XT Index kit. The pooled library was sequenced on the Illumina MiSeq using paired 300 bp reads at the University of Bristol Genomics Facility (Table S1, available in the online version of this article).

### Bioinformatics

The sequencing quality of each de-multiplexed fastq file was analysed using the FastQC software (http://www.bioinformatics.babraham.ac.uk/projects/fastqc/). The low-quality 3′ ends of all reads were trimmed. All forward reads were trimmed by 20 bp, and all reverse reads by 100 bp. All other processing steps were performed in Qiime [46]. The trimmed paired-end reads were joined before further processing and additionally filtered only allowing a minimum Phred quality score of Q20. Reads that could not be joined or were below the quality cut-off were excluded from the analysis. Chimeric sequences were removed using usearch 6.1. OTUs were picked *de novo* and clustered at 99 and 94.9% similarity for 18S and ITS2, respectively. Although an identity threshold of 97 % is widely used to delimitate species, a much stricter threshold is required for the 18S rRNA of algal communities that are dominated by Chlamydomonadales since several species in this group are very closely related and in some cases differ by only 1 bp over the length of the sequenced amplicon read. The threshold of 94.9 % for ITS2 is on par with several findings on the level of identity of algal ITS2 [47, 48] and also of soil fungal communities [49]. Taxonomic identities were assigned for representative sequences of each OTU using blast [50] and the reference database Silva [51] for 18S (extended with 223 additional sequences of cryophilic algae kindly provided by Dr Thomas Leya from the CCCryo – Culture Collection of Cryophilic Algae, Fraunhofer IZI-BB). For the assignment of ITS2 sequences, a custom database with the limited number of available reference sequences for (cryophilic) green algae was downloaded from NCBI (comprising 16 sequences) and added to the publicly available fungal UNITE database [52]. Singletons were removed from both the 18S and ITS2 data sets prior to further analysis. Since no ITS2 reference sequences are available for ice algae (e.g. *Ancylonema*, *Mesotaenium*), the representative sequences of the most abundant OTUs were submitted manually to the blast [50] web server to search NCBI for close hits to ice algal taxa. The community composition represents the relative abundance of taxa based on sequence frequency.

The most abundant taxa in the 18S data were subjected to oligotyping [39]. In order to reduce the complexity of the computational process, all 18S rRNA sequences that were assigned to the respective taxa were extracted individually and stored in separate fasta files. Low-quality ends (first and last 10 bp) were trimmed off in order to avoid inflated diversity due to sequencing errors prior to trimming all sequences to the same length of 320 bp using Fastx Trimmer (http://hannonlab.cshl.edu/fastx_toolkit/). Decomposition of individual oligotypes was stopped once the resolution could not be further improved. All analysis parameters for the individual taxa can be found in Table S2. All oligotypes were submitted to blast [50] to search for the most similar reference sequences.

### Microscopy

In order to qualitatively verify species assignment derived from sequencing, representative samples were imaged on a Zeiss Scope.A1 light microscope with Zeiss A-Plan ×40 and ×100 magnification objectives. Ice algal species were identified based on published morphological descriptions and photomicrographs of ice algae from other sites [6–8]. *Ancylonema* cells are larger and filamentous, whereas *Mesotaenium* cells are significantly smaller and generally unicellular or occasionally form very short chains [8]. Based on the sequence numbers, two samples dominated by *Ancylonema* and two samples dominated by *Mesotaenium* cells were evaluated and representative light micrographs were recorded for samples GrIS16_10 and GrIS16_18. A full assessment of cell counts in all samples was not feasible since samples were not appropriately preserved for microscopy, and thus degraded.

### Temperature data

Daily averaged temperature data for the period from 1 May to 31 August 2016 was obtained from three weather stations, which operate in close proximity to our sampling sites. Data for KAN_M (3 km away from site 3) and KAN_U (at site 1a) were provided by the Geological Survey of Denmark and Greenland (GEUS) [53], and data from S6 (2 km away from the base camp) were provided by the Institute for Marine and Atmospheric Research (IMAU) at Utrecht University.

## Results

### Community composition

**Fig. 2.**
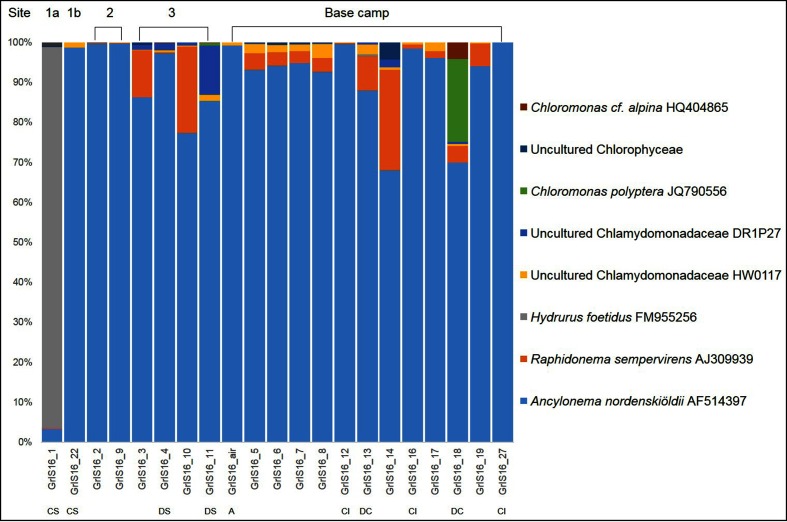
Algal community composition on the GrIS, with the eight most abundant taxa comprising >99 % of the total community at each sampling location. Full details can be found in Tables S3 and S4. The data presented here are based on the clustering of OTUs at 99 % similarity. The community composition based on the oligotyping approach can be found in Fig. 3. Locations are indicated above the histogram while sample types are shown below: CS, clean snow; DS, dirty snow; A, air; CI, clean ice; DC, dispersed cryoconite; all others represent dirty ice samples.

A total of 5 083 635 18S rRNA sequences passed the quality control, and 3 366 934 sequences could be assigned to algal taxa. The remaining sequences were predominantly assigned to fungi, as well as alveolata and rhizaria (data not shown). Only eight taxa made up >99 % of the entire algal community composition (Fig. 2 and Table S3; full community composition in Table S4). In all samples and independent of the sample type, the majority of the sequences were assigned to *A. nordenskiöldii* (between 66.0 and 97.7 %; 92 % of all sequences), with minor contributions of six Chlorophytes (Fig. 2, Table S3) with closest matches to *Raphidonema sempervirens*, two uncultured Chlamydomonadaceae, *Chloromonas* cf. *alpina* and *Chloromonas polyptera*. The only exception was the clean snow sample collected furthest from the base camp, which was mainly composed of the stramenopile *Hydrurus*. The pair of replicates that was collected in each of the two nearby sampling sites (GrIS_5/7 and GrIS_6/8; see Methods and Table 1) showed a very similar composition and therefore a high reproducibility and low spatial heterogeneity.

A total of 3 646 397 ITS2 sequences passed the quality control. Out of the 10 most abundant OTUs, nine were assigned to fungal taxa and one to the snow algae *R. sempervirens* (data not shown). The manual submission to the blast [50] web server resulted in the same findings. Since *A. nordenskiöldii* was by far the most abundant taxon in the 18S rRNA data set, being ten times more abundant than the most abundant fungal taxon, this likely means that its ITS2 could not be amplified with the primers used. This is the consequence of the lack of a reference sequence, which makes it impossible to check the suitability of the chosen primer pair *in silico*. The generation of more appropriate ITS2 reference sequences is mandatory in order for this marker to be successfully employed in community studies of cryophilic algae. Although ITS2 sequences were obtained for the less-abundant snow algae *R. sempervirens,* confirming some of the 18S findings, no sequences were retrieved for the more-abundant ice algae *A. nordenskiöldii*. ITS2 sequences, therefore, will not be further discussed in this study.

### Oligotyping

The four most abundant taxa, which made up 98 % of all 18S sequences, were chosen for oligotyping. These included *A. nordenskiöldii*, *R. sempervirens* and two uncultured Chlamydomonadaceae.

**Fig. 3.**
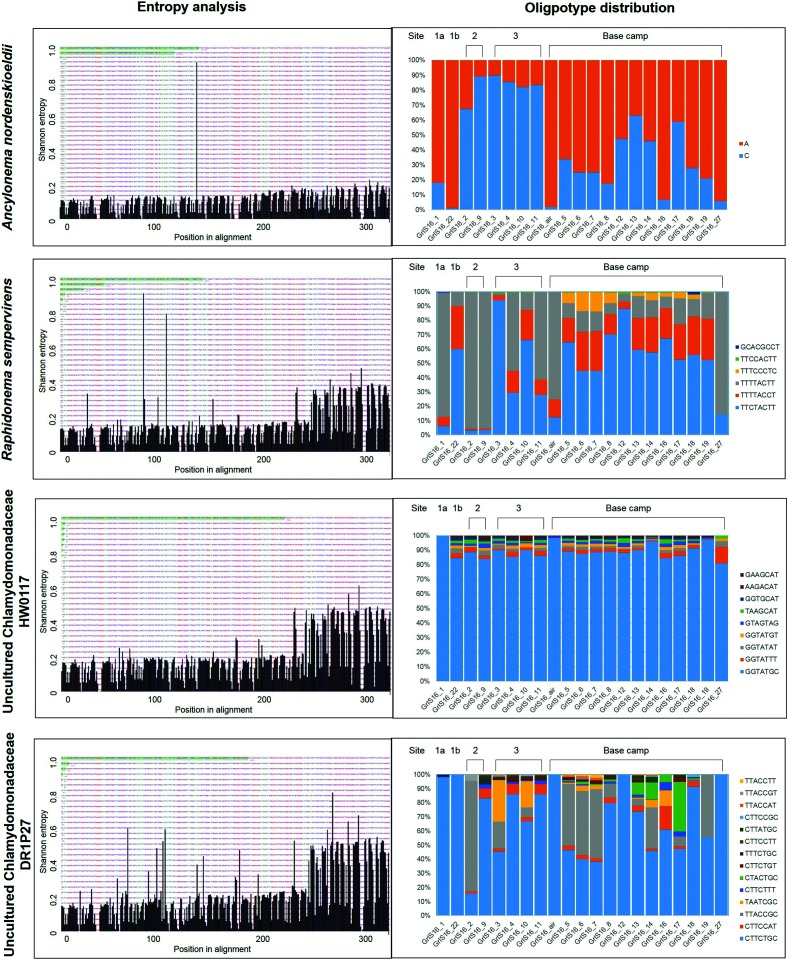
Graphs on the left show the most information-rich nucleotide position for the individual taxa, as revealed by the Shannon entropy analyses. Positions in the sequence alignment are on the x-axis, and the volume of Shannon entropy is on the y-axis. The entropy of each column in the nucleotide alignment was quantified [on a scale from 0 (none) to 1 (highest entropy)], and nucleotide positions with the highest entropy values were used for the oligotyping process. All the entropy peaks below ~0.2 can be regarded as sequencing noise and are likely not due to biological variation. The results of the Shannon entropy analyses were then used to derive distinct oligotypes, of which the distribution is displayed in the graphs on the right. The full details of the oligotype relative abundances can be found in Tables S5–S9. Oligotype names are derived from the nucleotide variations in the sequence alignment (Fig. S1).

Whereas *A. nordenskiöldii* was the taxon with by far the most sequences analysed (3 102 396), its 18S rRNA genes contained the lowest entropy and revealed only two oligotypes, which differed by only one nucleotide position (Fig. 3, Tables S2 and S5). Both oligotypes were present in all samples; however, their distribution differed considerably by location. Oligotype ‘C’ (oligotype names are derived from the nucleotide variations in the sequences) was more abundant in all samples from sites 2 and 3 (approximately 40 and 20 km away from the base camp, respectively). In contrast, oligotype ‘A’ was more abundant in all base camp samples (including the air sample), as well as the snow-covered site located approximately 100 km away from the base camp (Site 1a). Oligotype ‘A’ is 100 % identical with the reference sequence (Table S9) deposited in NCBI (AF514397). Oligotype ‘C’ shares 99.7 % similarity with *A. nordenskiöldii* and 99.4 % with *M. berggrenii* var. *alaskana* (JF430424.1).

*R. sempervirens* was the second most abundant taxa (139 646 sequences; 4.2 % of total sequences) and was characterized by six distinct oligotypes (Tables S2 and S6). All oligotypes shared 99–100 % similarity with the reference sequence of *R. sempervirens* (KM870611.1). Most oligotypes were present at all sites, whereas the fourth most abundant oligotype ‘TTTCCCTC’ was only found in the base camp samples.

The uncultured Chlamydomonadaceae HW0117 comprised 34 533 sequences, which were divided into eight oligotypes (Fig. 3, Tables S2 and S7). These shared between 93 and 99% similarity with the reference sequence (GU117575.1), which is closely related to *Chloromonas nivalis*. One oligotype (‘GGTATGC’) was by far the most abundant (89 % of all sequences). All other oligotypes were present in low abundances in most samples.

The second uncultured Chlamydomonadaceae, DR1P27, showed the highest entropy and oligotype number of 14, despite having the lowest number of sequences (31 593; Fig. 3, Tables S2 and S8). The blast [50] search revealed a 95–99 % similarity with *Chlamydomonas nivalis* (JQ790560.1) and 98–100 % similarity with *Ploeotila* sp. (GU117586.1; Table S9). Among the 14 oligotypes, oligotype ‘CTTCTGC’ made up 79 % of the sequences.

### Microscopy

**Fig. 4.**
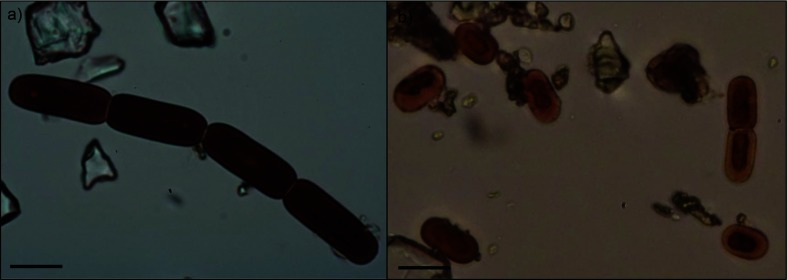
Representative light micrographs of ice algae in samples GrIS17_18 (a) and GrIS17_10 (b) showing the more abundant *Ancylonema* (a) and *Mesotaenium* (b) cells, respectively. Bars, 10 µm.

The sequencing results indicated that sample GrIS17_18 was dominated by *A. nordenskiöldii* oligotype ‘A’ (100 % similarity), and through microscopy we confirmed that this sample indeed contained a higher abundance of *Ancylonema* filaments (Fig. 4a). In contrast, sample GrIS17_10, which was characterized by a higher abundance of oligotype ‘C’ (99.7 and 99.4% similarities with *A. nordenskiöldii* and *M. berggrenii* var. *alaskana*, respectively), contained predominately single *Mesotaenium* cells (Fig. 4b).

### Temperature data

The daily temperature data obtained from the three weather stations showed a progressive decrease in average temperatures from the base camp (S6) to further inland (KAN_M and KAM_U) (Fig. S2). The daily averaged measurements indicated that the base camp (S6) experienced air temperatures that were 1 °C and 4 °C warmer than those documented at site 3 (KAN_M) and site 1a (KAN_U), respectively. In addition, during the period of the field campaign as well as the preceding 8 weeks, the air temperature at the base camp (S6) was predominantly above 0 °C, whereas temperatures measured at sites 3 and 1a (and likely also at site 2) were typically below freezing. These data can be used as a general proxy to assess the extent of the melting.

## Discussion

### Community composition

*A. nordenskiöldii* was the most abundant taxa, which matches previous findings from Greenland that were based on microscopic observations [6, 22]. The samples examined by Yallop *et al*. [6] were found to contain high abundances of *Cylindrocystis* spp. and *A. nordenskiöldii* and lower abundances of *Mesotaenium* spp. Based on our sequencing data, we found low abundances of *Cylindrocystis* spp. and *Mesotaenium* spp. (<0.02 and <0.5 % respectively, Table S4). However, the dominant *Ancylonema* oligotype ‘C’ shared almost the same sequence similarity with the *A. nordenskiöldii* and *M. berggrenii* var. *alaskana* reference sequences (99.7 and 99.4 %, respectively) over the 18S rRNA amplicon read sequenced. Thus, this oligotype could also represent a closely related *Mesotaenium* species, which is currently not present in the databases. Based on observations made using light microscopy (Fig. 4), the latter case is more likely. A high abundance of *Mesotaenium* cells could be observed in samples with more sequences assigned to the oligotype ‘C’. In contrast, *Ancylonema* cells were more abundant in samples with the majority of sequences matching oligotype ‘A’.

It is striking that *Ancylonema* and *Mesotaenium* were by far the dominant taxa in all samples and independent of the sample type. The exception was the clean snow sample collected at the most inland location site ~100 km away from the base camp (GrIS16_1). The community composition in this sample was almost entirely (96 %) made up by the Chrysophyceae taxon *Hydrurus*, previously found to be responsible for the less-often described ‘yellow snow’ [54]. This sample, however, contained the fewest sequences that could be assigned to algal taxa (sequences were mostly assigned to fungi; Table S1), a characteristic that may indicate a very low algal biomass.

All other taxa were snow algae (*Raphidonema*, uncultured Chlamydomonadaceae), which can commonly be found in the snow cover on glaciers or in permanent snow fields [24, 55, 56]. Thus, they are likely remnants from an earlier time point in the melt season, when the snow cover had started to melt and before the bare ice had been exposed. However, it is astonishing that the snow algal taxa were not more abundant in the other clean snow sample (GrIS16_22) and the two remnant dirty snow patches (GrIS16_4 and GrIS16_11). On glacial surfaces, a seasonal succession of algal communities with snow algae being dominant at the beginning of the melt season (or higher up the glacier in the snow cover) and ice algae towards the end (or in the lower bare ice area) of the melt season has been reported previously [10, 57]. This may not be the case for the interior of the GrIS, where the melting snow cover exhibiting optimal liquid water conditions for snow algae growth may be too short-lived, and thus explaining this disparity in community composition. The amount of available water is strongly influenced by slope, a morphological feature that the interior of the GrIS lacks. Furthermore, the nutrient conditions may not be suitable for the development of extensive snow algal blooms. As has been shown in several studies, physical processes (e.g. melting, slope dynamics) play a more influential role in controlling snow algal distribution and abundance than does snow chemistry [11, 24, 26, 55, 58, 59]. This is likely the case in the present study, in which no correlations were found between the ice algae distribution or abundance and any of the aqueous geochemical parameters measured (data not shown here).

### Oligotype distribution

The pitfall of high-throughput sequencing is often the limited read lengths, which requires the restriction to only a fragment of the chosen marker gene, and therefore a reduced taxonomic resolution. However, here we show that the chosen region of the 18S rRNA gene (v4-v5) is suitable to differentiate distinct taxa using oligotyping. The site-specific distribution patterns for the two oligotypes of *A. nordenskiöldii* likely suggest a different underlying ecology of the distinct oligotypes, which may be linked to differences in temperature and, in turn, the extent of the melting. The site-specific taxa distribution and the qualitative verification using light microscopy on representative samples clearly rule out the possibility that those oligotypes represent two operons within one individual. They more likely represent two distinct species that are subjected to competition and selection.

Oligotype ‘A’, which showed 100 % similarity with *A. nordenskiöldii*, was dominant in all base camp samples. In contrast, oligotype ‘C’, which is likely a *Mesotaenium* species, was the dominant oligotype in the samples from sites 2 and 3, which are further inland and at a higher elevation than the base camp sampling locations (1200–1400 vs 1000 m above sea-level; Table 1), and for which the temperature data indicates that they have likely experienced a later onset of melting (Fig. S2). Thus, this could be explained by a transition of the dominant species with *Mesotaenium* showing a higher abundance at the onset of melting and *Ancylonema* increasing in abundance with progressing melting.

In contrast, the high similarity of the six *R. sempervirens* oligotypes (99–100 %; Tables S6 and S9) and the approximately even distribution and similar abundances of the oligotypes across all base camp samples suggests that these oligotypes represent intragenomic variants of this species. The same applies to the uncultured Chlamydomonadaceae HW0117, which was mostly represented by one oligotype. All other oligotypes co-occurred evenly in all samples with low abundances (Fig. 3, Tables S2 and S7), which suggests that these might represent different copies of the 18S rRNA gene within one individual, rather than distinct species. In contrast, the oligotypes of the uncultured Chlamydomonadaceae DR1P27 showed large variations across all samples (Fig. 3, Tables S2 and S8). Together with low similarities with the reference sequence (as low as 95 %; Table S9), this suggests a variety of unknown and low-abundant snow algal species.

In conclusion, we show that an OTU-based approach is not sufficient for the evaluation of the extremely low ice algal diversity on the GrIS. Oligotyping revealed hidden diversity that could not be detected by conventional clustering of OTUs and taxonomic classification. *A. nordenskiöldii* and a *Mesotaenium* species were by far the dominant taxa. *Ancylonema* was more abundant at the onset of the melt season while *Mesotaenium* dominated towards the end. The site-specific distribution of the oligotypes may be linked to the extent of the melting, with a transition from *Mesotaenium* to *Ancylonema* with melt progression over time. Our data indicate that characterizing the links between community composition and the different stages of the melt season, along with other possible effects on melt rates (i.e. inputs and proportion of mineral dust, black carbon or water content of ice and snow) have to be the focus of future studies, as only by doing so can the role of pigmented algae in changing the albedo of the GrIS be evaluated.

## Data bibliography

1. DNA sequences have been deposited to the European Nucleotide Archive (ENA) under accession number ERP104425 and can be accessed under https://figshare.com/s/2c10e295e7b6f20bb16a. All other data is available in the supplementary data files.

## Supplementary Data

846 Supplementary File 1
