# Supplementary material for: The diversity of ice algal communities on the Greenland Ice Sheet as revealed by oligotyping
Source: Microb Genom. 2018 Mar 16;4(3):e000159. doi: 10.1099/mgen.0.000159 (PMC5885011; doi:10.1099/mgen.0.000159)

Supplementary material

The diversity of ice algal communities on the Greenland Ice Sheet as revealed by oligotyping

Supplementary tables: 9
Supplementary figures: 2

Supplementary Table 1: Number of 18S rRNA sequences before and after quality filtering, as well as the number and proportion (%) of sequences assigned to algal taxa (the remaining sequences were mostly assigned to Fungi as well as Alveolata, Rhizaria, etc.; data not shown).

Sample	Site	No. of sequences before quality filtering	No. of sequences after quality filtering	No. and % of sequences assigned to algae
**GrIS16_1**	1a	146099	144073	693 (0.5%)
**GrIS16_22**	1b	243230	240351	173475 (72.2%)
**GrIS16_2**	2	275392	277644	2771811 (97.9%)
**GrIS16_9**	2	250717	248118	185536 (74.8%)
**GrIS16_3**	3	295439	291935	187219 (64.1%)
**GrIS16_4**	3	264845	261526	165560 (63.3%)
**GrIS16_10**	3	230620	227867	143220 (62.9%)
**GrIS16_11**	3	270128	266856	158082 (59.2%)
**GrIS16_air**	base camp	107074	106643	94889 (89.0%)
**GrIS16_5**	base camp	189871	187632	116807 (62.3%)
**GrIS16_6**	base camp	249045	246194	161330 (65.5%)
**GrIS16_7**	base camp	251583	248691	169012 (68.0%)
**GrIS16_8**	base camp	208418	206044	129523 (62.9%)
**GrIS16_12**	base camp	319713	315965	193428 (61.2%)
**GrIS16_13**	base camp	404161	399334	242681 (60.8%)
**GrIS16_14**	base camp	285202	281855	106056 (37.6%)
**GrIS16_16**	base camp	303049	299676	253856 (84.7%)
**GrIS16_17**	base camp	211482	209216	140781 (67.3%)
**GrIS16_18**	base camp	182810	180638	102310 (56.6%)
**GrIS16_19**	base camp	219933	217536	170931 (78.6%)
**GrIS16_27**	base camp	234299	231674	193901 (83.7%)
**Total**		5143110	5089468	3361101 (66.0%)

Supplementary Table 2: Parameters used for the oligotyping analyses of the most abundant taxa in the GrIS samples.

	Individual sequences analysed	Number of entropy locations	Base locations	Min % abundance of oligotype in at least one sample	Min substantive abundance of an oligotype	No. of oligotypes	Total purity score [%]
**Ancylonema nordenskiöldii AF514397**	3,102,396	1	133	1	50	2	99
**Raphidonema sempervirens AJ309939**	139,646	8	66, 81, 82, 83, 96, 103, 104, 149	1	50	6	93
**Uncultured Chlamydomonadaceae HW0117 GU117575**	34,533	7	159, 170, 171, 192, 195, 279, 289	1	10	8	85
**Uncultured Chlamydomonadaceae DR1P27 GU117577**	31,592	7	67, 87, 103, 134, 265, 280, 290	1	10	14	41

Supplementary Table 3: Algal community composition comprising the 8 most abundant taxa. Values are reported in % of overall relative abundance inferred from sequence frequency and clustering of OTUs at 99% similarity. Table shows taxa with >0.1% relative abundance.

	**Site 1a**	**Site 1b**	**Site 2**		**Site 3**			
**Taxon**	GrIS16 1	GrIS16 22	GrIS16 2	GrIS16 9	GrIS16 3	GrIS16 4	GrIS16 10	GrIS16 11
Ancylonema nordenskiöldii AF514397	3.4	**98.6**	**99.7**	**99.6**	**86**	**97.2**	**77.3**	**85.3**
Raphidonema sempervirens AJ309939	0.3	0	0.2	0.1	11.8	0.2	21.7	0
Hydrurus foetidus FM955256	**96.0**	0	0	0	0	0	0	0
Uncultured Chlamydomonadaceae HW0117 GU117575	0	1.3	0.1	0.2	0.1	0.6	0.2	1.6
Uncultured Chlamydomonadaceae DR1P27GU117577	0	0	0.1	0	1.2	1.7	0.8	12.3
Chloromonas polyptera JQ790556	0	0	0	0	0	0.1	0	0.6
Uncultured Chlorophyceae	0.2	0	0	0	0.6	0	0	0
Chloromonas cf. alpina HQ404865	0	0	0	0	0	0.1	0	0.2

Supplementary Table 3 continued.

	**Base camp**												
**Taxon**	GrIS16 air	GrIS16 5	GrIS16 6	GrIS16 7	GrIS16 8	GrIS16 12	GrIS16 13	GrIS16 14	GrIS16 16	GrIS16 17	GrIS16 18	GrIS16 19	GrIS16 27
Ancylonema nordenskiöldii AF514397	**99.2**	**92.7**	**93.5**	**94.2**	**92.2**	**99.5**	**88**	**66**	**98.4**	**95.9**	**66.9**	**94**	**99.2**
Raphidonema sempervirens AJ309939	0	4.1	3.3	2.9	3.5	0.3	8.5	**24.3**	1.1	1.7	4	5.7	0
Hydrurus foetidus FM955256	0	0	0	0	0	0	0.5	0	0	0	0	0	0
Uncultured Chlamydomonadaceae HW0117 GU117575	0.8	2.3	1.9	1.7	3.5	0.1	2.5	0.6	0.5	2.2	0.5	0.3	0
Uncultured Chlamydomonadaceae DR1P27 GU117577	0	0.2	0.1	0.1	0.2	0	0.5	2	0	0	0.5	0	0
Chloromonas polyptera JQ790556	0	0	0	0	0	0	0	0	0	0	**19.8**	0	0
Uncultured Chlorophyceae	0	0.2	0.5	0.4	0.2	0	0	4.1	0	0	0	0	0
Chloromonas cf. alpina HQ404865	0	0	0	0	0	0	0	0	0	0	4.0	0	0

Supplementary Table 4: Table shows full algal community composition in % of overall relative abundance inferred from sequence frequency and clustering of OTUs at 99% similarity.

Taxon	GrIS16 1	GrIS16 22	GrIS16 2	GrIS16 9	GrIS16 3	GrIS16 4	GrIS16 10	GrIS16 11	GrIS16 air	GrIS16 5	GrIS16 6	GrIS16 7	GrIS16 8	GrIS16 12	GrIS16 13	GrIS16 14	GrIS16 16	GrIS16 17	GrIS16 18	GrIS16 19	GrIS16 21	GrIS16 27
**Ancylonema nordenskioeldii**	2.91	98.48	98.85	99.08	84.62	96.68	76.15	84.22	88.05	91.96	93.06	93.91	91.48	99.39	87.27	64.71	98.09	95.23	63.84	93.96	76.87	99.12
**Botrydiopsis callosa**	0	0	0	0	0	0	0	0	0	0	0	0	0	0	0	0	0	0	0.44	0	0	0
**Botrydiopsis constricta**	0	0	0.2	0.01	0.08	0	0.28	0.01	0	0	0	0	0	0.01	0.01	0.08	0	0	4.17	0	0.2	0
**Chlamydomonadaceae sp CCCryo 086a-99 GU117586**	0	0	0.01	0	0.09	0	0.04	0	0	0.01	0.01	0.01	0.01	0	0	0.05	0	0	0	0	0	0
**Chlamydomonas cf proboscigera CCCryo 217-05**	0	0	0	0	0	0	0	0	0	0	0	0	0	0	0.14	0.01	0	0.01	0.02	0	0	0
**Chlorellidium tetrabotrys**	0	0	0	0	0	0	0	0	0	0	0	0	0	0	0	0	0	0	0	0	0	0
**Chlorococcalean sp AF514412**	0	0	0	0	0	0	0	0	0	0	0	0	0	0	0	0	0	0	0	0	0	0
**Chloroidium saccharophilum**	0	0	0	0	0	0	0	0	0	0	0	0	0	0	0	0	0	0	1.94	0	0	0
**Chloromonas cf alpina CCCryo 033-99**	0	0	0	0.03	0.03	0.12	0	0.19	0	0	0	0	0	0	0	0	0	0.01	3.87	0	0	0
**Chloromonas cf insignis CCCryo 090-99 GU117585**	0	0	0.17	0	0.03	0	0.01	0	0	0	0	0	0	0	0.01	0	0	0	0	0	0	0
**Chloromonas cf rostafisnkii CCCryo 025-99**	0	0	0	0	0	0	0	0	0	0	0	0	0	0	0	0	0	0	0	0	0	0
**Chloromonas cf schussnigii CCCryo 082-99GU117584**	0	0	0.01	0	0	0	0	0	0	0	0	0	0	0	0	0	0	0	0	0	0	0
**Chloromonas nivalis CCCryo 005-99 AF514409**	0	0	0	0	0	0	0	0	0	0	0	0	0	0	0	0	0	0	0	0	0	0
**Chloromonas nivalisAF517088**	0	0	0	0	0	0	0	0	0	0	0	0	0	0	0	0	0	0	0.03	0	0	0
**Chloromonas pichinchae**	0	0	0	0	0	0	0	0	0	0	0	0	0	0	0	0	0	0	0.02	0	0	0
**Chloromonas polyptera JQ790556**	0	0.01	0	0	0	0.08	0.01	0.64	0.01	0	0	0	0	0.01	0.02	0.01	0.02	0.01	19.07	0	0.01	0
**Chloromonas spec CCCryo 273-06**	0	0	0.03	0	0	0	0	0	0	0	0	0	0	0	0.02	0	0	0	0	0	0	0
**Chloromonas tughillensis**	0	0	0	0	0	0	0	0	0	0	0	0	0	0	0	0	0	0	0	0	0	0
**Chloromonas tughillensis UTEX SNO 88**	0	0	0	0	0	0	0	0	0	0	0	0	0	0	0	0	0	0	0.11	0	0	0
**Chloromonas typhlos SAG2686**	0	0	0	0	0	0	0	0	0	0	0	0	0	0	0	0	0	0	0	0	0	0
**Chrysophyceae sp. I76**	0	0	0	0	0	0	0	0	0	0	0	0	0.01	0	0	0	0	0	0	0	0	0.02
**Coccomyxa pringsheimii**	0	0	0	0	0	0	0	0	0	0	0	0	0	0	0	0	0	0	0	0	0	0
**Coccomyxa sp. Brodo**	0	0	0	0	0	0	0	0	0	0	0	0	0	0	0	0	0	0	0	0	0	0

**Coenocystis inconstans**	0	0	0	0	0.02	0.01	0.03	0.04	0	0.01	0	0	0.01	0	0.02	0.01	0	0	0.02	0	0	0
**Coleochaete orbicularis**	0	0	0	0	0	0	0	0	0	0	0	0	0	0	0	0	0	0	0	0	0	0
**Cosmarium botrytis**	0	0	0	0	0	0	0	0	0	0	0	0	0	0	0	0	0	0	0	0	0	0
**Cosmarium contractum**	0	0	0	0.02	0	0.01	0	0	0	0	0	0	0	0	0	0	0	0.01	0	0	0	0
**Cosmarium subprotumidum**	0	0	0	0	0	0	0	0	0	0	0	0	0	0	0	0	0	0	0	0	0	0
**Cylindrocystis brebissonii var. deserti**	0	0	0.01	0	0	0	0	0	0	0	0	0	0	0	0	0	0	0	0	0	0	0
**Cylindrocystis sp. M3015**	0	0	0	0	0	0	0	0	0	0.02	0.02	0.01	0.01	0	0	0.1	0	0.03	0.01	0	0.02	0
**Heterolobosea sp. OSA**	0	0	0	0	0	0	0.02	0	0	0	0	0	0	0	0	0	0	0	0	0	0	0
**Heterothrix debilis**	0	0	0	0	0	0	0	0	0	0	0	0	0	0	0	0	0	0	0	0	0	0
**Hormidiella attenuata**	0	0	0.01	0	0	0	0	0	0	0	0	0	0	0	0	0	0	0	0	0	0	0
**Hydrurus foetidus**	80.46	0	0	0	0	0	0	0	0.27	0	0	0	0	0	0.48	0.02	0	0.03	0.01	0	0.1	0.01
**Klebsormidium flaccidum**	0	0	0	0	0	0	0	0	0	0	0	0	0	0	0	0	0	0	0	0	0	0
**Mesotaenium berggrenii var alaskana**	0	0.03	0.17	0.31	0.2	0.28	0.12	0.15	0	0.04	0.07	0.05	0.05	0.09	0.06	0.06	0.08	0.24	0.07	0	0	0.02
**Mesotaenium cf. chlamydosporum M-2155**	0	0	0	0	0	0	0	0	0	0	0	0	0	0	0	0	0	0	0	0	0	0
**Mesotaenium sp. AG-2009-1**	0	0	0	0	0	0	0	0	0	0	0	0	0	0	0	0	0	0	0.03	0	0	0
**Microglena polar subclade**	0.32	0	0	0	0	0	0	0	0.01	0	0	0	0	0	0.07	0.01	0	0	0.01	0	0.04	0
**Mychonastes homosphaera**	0	0	0	0	0	0	0	0	0	0	0	0	0	0	0	0	0	0	0	0	0	0
**Myrmecia bisecta**	0	0	0	0	0	0	0	0	0	0	0	0	0	0	0	0	0	0	0.03	0	0	0
**Neocystis brevis**	0	0	0	0	0	0	0	0	0	0	0	0	0	0	0	0	0	0	0	0	0	0
**Neovahlkampfia damariscottae**	0	0	0	0	0.07	0	0.06	0	0	0	0	0	0	0	0.02	0.15	0	0	0	0	0	0
**Nephroselmis rotunda**	0	0	0	0	0.02	0	0.05	0	0	0.01	0	0.01	0	0	0.02	0.01	0	0	0.01	0	0	0
**No blast hit**	0.34	0.01	0.05	0.09	0.29	0.03	0.33	0.02	0.03	0.1	0.11	0.11	0.12	0.01	0.16	0.41	0.02	0.1	0.11	0.02	0.29	0.04
**Ochromonadaceae environmental sample**	0	0	0	0	0	0	0	0	0	0	0	0	0	0	0	0	0	0	0	0	0	0
**Ostreococcus tauri**	0	0	0	0	0	0	0	0	0	0	0	0	0	0	0	0	0	0	0	0	0	0
**Penium margaritaceum**	0	0	0	0	0	0	0	0	0	0	0	0	0	0	0	0	0	0	0	0	0	0
**Porphyridium sp. MBIC10451**	0	0	0	0	0	0	0	0	0	0	0	0	0	0	0	0	0	0	0	0	0	0
**Prasiola fluviatilis**	0	0	0	0	0	0	0	0	0	0	0	0	0	0	0	0.01	0	0	0	0	0	0
**Prototheca cutis**	0	0	0	0	0	0	0	0	0	0	0	0	0	0	0	0	0	0	0.12	0	0	0
**Pseudochlorella pringsheimii**	0	0	0	0	0	0	0	0	0	0	0	0	0	0	0	0	0	0	0	0	0	0

**Pseudopediastrum kawraiskyi**	0	0	0	0	0	0	0	0	0	0	0	0	0	0	0	0	0	0	0	0	0	0
**Raphidonema sempervirens AJ309939**	0.28	0.01	0.17	0.13	11.6	0.17	21.34	0.05	0.01	4.08	3.29	2.93	3.48	0.34	8.48	23.88	1.1	1.72	3.84	5.64	20.41	0
**Saprolegnia sp. SAP1**	0.01	0	0	0	0.49	0	0.02	0	0	0.02	0.01	0.01	0.03	0	0	0.28	0.02	0	0	0	0	0
**Sphaerozosma granulatum**	0	0	0	0	0	0	0	0	0	0	0	0	0	0	0	0	0	0	0	0	0	0
**Staurodesmus mucronatus**	0.01	0	0	0	0	0	0	0	0	0.36	0.01	0.01	0.25	0	0.01	0.7	0	0.04	0.01	0	0	0
**Stichococcus bacillaris**	0	0	0	0	0	0	0	0	0	0	0	0	0	0	0	0	0	0	0	0	0	0
**Stichococcus bacillaris**	0	0	0	0	0	0	0	0	0	0	0	0	0	0	0	0	0	0	0.99	0	0	0
**Stichococcus jenerensis**	0	0	0	0	0	0	0	0	0	0	0	0	0	0	0	0	0	0	0	0	0	0
**Stichococcus mirabilis**	0	0	0	0	0.01	0	0.02	0	0	0	0	0	0	0	0	0	0	0	0.02	0	0	0
**Trebouxia usneae**	0	0	0	0	0	0	0	0	0	0.04	0	0	0	0	0	0	0	0	0	0	0	0
**uncultured Banisveld eukaryote**	0	0	0	0	0	0	0	0	0	0	0	0	0	0	0	0	0	0	0	0	0	0
**Uncultured Chlamydomonadaceae clone HW0117 Cr nivalis**	0.02	1.45	0.11	0.25	0.12	0.68	0.2	1.67	0.62	2.43	2.05	1.75	3.67	0.12	2.58	0.61	0.5	2.27	0.44	0.29	0.65	0.03
**Uncultured Chlamydomonadaceae GU117577**	0.03	0	0.05	0.04	1.16	1.83	0.77	12.85	0	0.18	0.13	0.1	0.25	0	0.46	1.98	0.01	0.04	0.46	0.01	1.14	0.01
**uncultured Closteriaceae**	0	0	0.07	0	0.01	0.02	0	0.01	0	0.01	0.01	0.01	0	0	0.01	0.01	0.02	0.08	0.02	0	0	0
**uncultured Closteriaceae**	0	0	0	0	0	0	0	0	0	0	0	0	0	0	0	0	0	0	0	0	0	0
**uncultured dinoflagellate**	0	0	0	0	0	0	0	0	0	0	0	0	0	0	0	0	0	0	0	0	0	0
**uncultured Eimeriidae**	0.13	0	0	0	0.61	0	0	0	0.01	0.22	0.52	0.45	0.18	0	0	4	0	0	0	0.02	0	0
**Uncultured marine stramenopile**	0.02	0	0.01	0.01	0.21	0.01	0.01	0.01	0	0.09	0.15	0.13	0.35	0.01	0.02	1.26	0.01	0.07	0.03	0.01	0	0.72
**uncultured Prasinophyceae**	0	0	0	0	0.18	0	0.4	0	0	0	0	0	0	0	0	0	0	0	0	0	0.01	0
**Uncultured stramenopile**	0	0	0	0	0	0	0	0	0	0	0	0	0	0	0	0	0	0	0	0	0	0
**uncultured Zygnematales**	0	0	0	0	0	0	0	0	0	0	0	0	0	0	0	0	0	0	0	0	0	0
**Vahlkampfia avara**	0	0	0	0	0	0	0	0	0	0	0	0	0	0	0	0.01	0	0	0	0	0	0
**Xanthophyceae sp. IX3**	0	0	0	0	0	0	0	0	0	0	0	0	0	0	0	0	0	0	0.12	0	0	0
**Zygnema circumcarinatum**	0	0	0.02	0.01	0.1	0.06	0.07	0.07	0	0.01	0.01	0.01	0.02	0	0.02	0	0.01	0.02	0.02	0	0	0.01
**Zygnema cylindricum**	0	0	0	0	0	0	0	0	0	0	0	0	0	0	0	0	0	0	0	0	0	0

Supplementary Table 5: Oligotype distribution of *Ancylonema nordenskiöldii* in % of overall relative abundance. Oligotype names are derived from the nucleotide variations in the sequence alignment.

Sample	Site	C	A
**GrIS16_1**	1a	18.2	81.8
**GrIS16_22**	1b	1.2	98.8
**GrIS16_2**	2	67.5	32.5
**GrIS16_9**	2	89.2	10.8
**GrIS16_3**	3	89.8	10.2
**GrIS16_4**	3	85.4	14.6
**GrIS16_10**	3	82.0	18.0
**GrIS16_11**	3	83.7	16.3
**GrIS16_air**	base camp	1.9	98.1
**GrIS16_5**	base camp	34.0	66.0
**GrIS16_6**	base camp	25.2	74.8
**GrIS16_7**	base camp	25.1	74.9
**GrIS16_8**	base camp	17.7	82.3
**GrIS16_12**	base camp	47.5	52.5
**GrIS16_13**	base camp	63.2	36.8
**GrIS16_14**	base camp	46.2	53.8
**GrIS16_16**	base camp	6.9	93.1
**GrIS16_17**	base camp	59.0	41.0
**GrIS16_18**	base camp	28.0	72.0
**GrIS16_19**	base camp	20.9	79.1
**GrIS16_27**	base camp	5.8	94.2

Supplementary Table 6: Oligotype distribution of *Raphidonema sempervirens* in % of overall relative abundance. Oligotype names are derived from the nucleotide variations in the sequence alignment.

Sample	Site	TTCTACTT	TTTTACCT	TTTTACTT	TTTCCCTC	TTCCACTT	GCACGCCT
**GrIS16_1**	1a	6.4	6.4	87.2	0	0	0
**GrIS16_22**	1b	60.0	30.0	10.0	0	0	0
**GrIS16_2**	2	3.4	0.8	95.3	0	0.4	0
**GrIS16_9**	2	3.7	0.9	95.4	0	0	0
**GrIS16_3**	3	94.1	3.5	1.6	0	0.8	0
**GrIS16_4**	3	29.5	15.1	55.0	0	0.4	0
**GrIS16_10**	3	66.4	20.6	12.3	0	0.6	0
**GrIS16_11**	3	28.0	10.7	61.3	0	0	0
**GrIS16_air**	base camp	12.5	12.5	75.0	0	0	0
**GrIS16_5**	base camp	64.9	16.8	10.4	7.3	0.6	0
**GrIS16_6**	base camp	44.7	27.2	14.6	13.2	0.3	0
**GrIS16_7**	base camp	44.8	27.8	13.7	13.3	0.4	0
**GrIS16_8**	base camp	70.1	14.1	7.9	7.3	0.6	0
**GrIS16_12**	base camp	88.3	4.8	5.6	0.2	1.1	0
**GrIS16_13**	base camp	59.6	22.1	15.1	2.6	0.6	0
**GrIS16_14**	base camp	57.7	24.3	12.2	5.2	0.7	0
**GrIS16_16**	base camp	67.4	21.0	8.2	2.8	0.6	0
**GrIS16_17**	base camp	52.5	24.9	18.0	4.3	0.3	0
**GrIS16_18**	base camp	55.9	26.8	12.2	3.1	0.3	1.8
**GrIS16_19**	base camp	52.5	28.7	18.1	0.2	0.5	0
**GrIS16_27**	base camp	14.3	0	85.7	0	0	0

Supplementary Table 7: Oligotype distribution of the uncultured *Chlamydomonadaceae* HW0117 in % of overall relative abundance. Oligotype names are derived from the nucleotide variations in the sequence alignment.

Sample	Site	GGTATGC	GGTATTT	GGTATAT	GGTATGT	GTAGTAG	TAAGCAT	GGTGCAT	GAAGCAT
**GrIS16_1**	1a	100	0	0	0	0	0	0	0
**GrIS16_22**	1b	84.9	3.3	3.1	2.0	2.4	1.8	2.0	0.5
**GrIS16_2**	2	89.0	2.8	0.7	0.7	1.4	2.8	1.4	1.4
**GrIS16_9**	2	84.2	2.2	3.3	1.8	4.0	1.6	0.9	2.0
**GrIS16_3**	3	90.2	0.9	2.7	0.9	2.2	1.8	1.3	0.0
**GrIS16_4**	3	85.5	3.6	2.0	2.2	3.0	1.5	1.5	0.5
**GrIS16_10**	3	90.1	1.8	0	2.9	3.6	0.7	0.7	0
**GrIS16_11**	3	86.3	3.3	1.9	2.2	2.2	2.1	1.4	0.6
**GrIS16_air**	base camp	98.2	0	0.2	0	1.5	0.2	0.0	0
**GrIS16_5**	base camp	89.3	2.0	1.8	1.9	2.1	1.5	1.0	0.4
**GrIS16_6**	base camp	87.6	2.5	2.2	1.7	2.3	1.5	1.3	1.0
**GrIS16_7**	base camp	88.9	2.1	1.7	2.0	2.3	1.3	1.1	0.6
**GrIS16_8**	base camp	89.0	2.3	1.6	1.4	2.5	1.5	1.3	0.5
**GrIS16_12**	base camp	88.4	2.2	0.4	1.3	2.7	3.1	0.9	0.9
**GrIS16_13**	base camp	90.0	1.7	1.7	1.5	2.2	1.2	1.1	0.6
**GrIS16_14**	base camp	96.4	0.3	0.2	0.6	1.7	0.6	0	0.2
**GrIS16_16**	base camp	84.7	3.3	2.1	2.3	2.6	2.9	1.8	0.3
**GrIS16_17**	base camp	86.3	3.2	2.0	2.2	2.8	1.5	1.3	0.9
**GrIS16_18**	base camp	91.4	2.1	0.2	1.1	2.6	1.1	1.1	0.4
**GrIS16_19**	base camp	97.1	0.2	0.4	0	1.4	0	0.2	0.6
**GrIS16_27**	base camp	81.1	11.3	3.8	1.9	0	1.9	0	0

Supplementary Table 8: Oligotype distribution of the uncultured *Chlamydomonadaceae* DR1WP27 in % of overall relative abundance. Oligotype names are derived from the nucleotide variations in the sequence alignment.

Sample	Site	CTTCTGC	CTTCCAT	TTACCGC	TAATCGC	CTTCTTT	CTACTGC	CTTCTGT	TTTCTGC	CTTCCTT	CTTATGC	CTTCCGC	TTACCAT	TTACCGT	TTACCTT
**GrIS16_1**	1a	100	0	0	0	0	0	0	0	0.0	0	0	0	0	0
**GrIS16_22**	1b	100	0.0	0	0	0	0	0	0	0	0	0	0	0	0
**GrIS16_2**	2	15.9	1.4	78.3	0	0	0	0	0	0	0	0	0	4.3	0
**GrIS16_9**	2	83.1	7.0	0	0	2.8	0	0	5.6	1.4	0	0	0	0	0
**GrIS16_3**	3	45.4	2.2	19.0	29.3	0.6	0	0.5	0.7	0.5	0	0.3	0.6	0.6	0.3
**GrIS16_4**	3	86.1	7.1	0	0	1.7	0	2.1	1.7	1.3	0	0	0	0	0
**GrIS16_10**	3	67.0	2.9	6.9	18.5	0.8	0.1	1.3	0.8	0.6	0	1.0	0.1	0	0
**GrIS16_11**	3	86.2	7.2	0	0	2.4	0	1.6	1.6	0.9	0	0.1	0	0	0
**GrIS16_air**	base camp	100	0	0	0	0	0	0	0	0	0	0	0	0	0
**GrIS16_5**	base camp	46.7	2.9	43.8	1.0	1.0	1.0	1.4	0.5	0	0	0	1.4	0	0.5
**GrIS16_6**	base camp	40.1	2.9	45.4	3.9	1.0	1.9	1.0	0.5	0	0	0.5	1.9	0	1.0
**GrIS16_7**	base camp	38.3	2.3	49.1	2.9	0	0.6	2.3	0	0.6	0	0	1.7	0	2.3
**GrIS16_8**	base camp	79.8	4.0	9.7	0	1.6	1.6	1.2	1.6	0.6	0	0	0	0	0
**GrIS16_12**	base camp	100	0	0	0	0	0	0	0	0	0	0	0	0	0
**GrIS16_13**	base camp	74.0	4.1	4.5	1.3	2.0	8.7	1.0	1.0	0.6	2.3	0.2	0.1	0.2	0.2
**GrIS16_14**	base camp	46.0	1.5	29.3	5.3	0.3	12.1	0.3	0.1	0	3.5	0.7	0.5	0.2	0.1
**GrIS16_16**	base camp	61.1	16.7	0	11.1	5.6	5.6	0	0	0	0	0	0	0	0
**GrIS16_17**	base camp	47.4	1.8	7.0	0	3.5	35.1	3.5	0	0	1.8	0	0	0	0
**GrIS16_18**	base camp	91.5	3.9	0.4	0	0.6	0.8	0.4	0.4	0	0	1.9	0	0	0
**GrIS16_19**	base camp	55.6	0	44.4	0	0	0	0	0	0	0	0	0	0	0
**GrIS16_27**	base camp	100	0	0	0	0	0	0	0	0	0	0	0	0	0

Supplementary Table 9: Total abundance of each oligotype, their fasta sequences and the closest BLAST matches. Oligotype names are derived from the nucleotide variations in the sequence alignment.

Oligotyping				Best BLAST hit				
**Taxon**	**Oligotype**	**Total abundance**	**Fasta sequence**	**Accession number**	**Description**	**Query cover**	**e-value**	**Identity**
**Ancylonema**	A	1,744,812	CCAGCTCCAATAGCGTATATTTAAGTTGTTGCAGTTA<br>AAAAGCTCGTAGTTGGATTTTGGGTTGGTGTGGTCG<br>GTCTGCTTTCTAGTTGAACTGGCTACTCCATCCTTCT<br>TGCCGGGGACGCGTTCTGGCCTTAATTGGCTGGGA<br>CGCGGAGTCGGCGATGTTACTTTGAAAAAATTAGAG<br>TGTTCAAAGCAGGCCTACGCTCTGAATACATTAGCA<br>TGGAATAACGTGATAGGACTCTGGTCCTATTGTGTT<br>GGTCTTCGGGACCGGAGTAATGATTAATAGGGACA<br>GTTGGGGGCATTCGTATTTCATTGTCAGAGGT	AF514397.2	Ancylonema nordenskiöldii strain CCCryo BS_0001-2000 18S ribosomal RNA gene, partial sequence	100%	3e-165	100%
	C	1,397,049	CCAGCTCCAATAGCGTATATTTAAGTTGTTGCAGTTA<br>AAAAGCTCGTAGTTGGATTTTGGGTTGGTGTGGTCG<br>GTCTGCTTTCTAGTTGAACTGGCTACTCCATCCTTCT<br>TGCCGGGGACGCGTTCTGGCCTTCATTGGCTGGGA<br>CGCGGAGTCGGCGATGTTACTTTGAAAAAATTAGAG<br>TGTTCAAAGCAGGCCTACGCTCTGAATACATTAGCA<br>TGGAATAACGTGATAGGACTCTGGTCCTATTGTGTT<br>GGTCTTCGGGACCGGAGTAATGATTAATAGGGACA<br>GTTGGGGGCATTCGTATTTCATTGTCAGAGGT	AF514397.2	Ancylonema nordenskiöldii strain CCCryo BS_0001-2000 18S ribosomal RNA gene, partial sequence	100%	1e-163	99%
				JF430424.1	Mesotaenium berggrenii var. alaskana	100%	6e-162	99%
**Raphidonema sempervirens**	TTCTACTT	96,107	CCAGCTCCAATAGCGTATATTTAAGTTGCTGCAGTTA<br>AAAAGCTCGTAGTTGGATTTCGGATGGGTTCCGTCG<br>GTCCGCCGTCTCGGTGTGCACTGACGGCGCCTATC<br>TTGCTGTCGGGGACGGGCTCCTGGGCTTCACTGTC<br>CGGGACTCGGAGTCGACGAGGTTACTTTGAGTAAAT<br>TAGAGTGTTCAAAGCAGGCCTACGCTCTGAATACAT<br>TAGCATGGAATAACACGATAGGACTCTGGCCTATCT<br>TGTTGGTCTGTAGGACCGGAGTAATGATTAAGAGGG<br>ACAGTCGGGGGCATTCGTATTTCATTGTCAGAG	KM870650.1	Uncultured eukaryote clone TE107A 18S ribosomal RNA gene, complete sequence	100%	3e-165	100%
				AF514410.2	Raphidonema sempervirens strain CCCryo 011a-99 18S ribosomal RNA gene, partial sequence	100%	1e-163	99%
	TTTTACCT	30,283	CCAGCTCCAATAGCGTATATTTAAGTTGCTGCAGTTA<br>AAAAGCTCGTAGTTGGATTTCGGATGGGTTCCGTCG<br>GTCCGCCGTTTCGGTGTGCACTGACGGCGCCCATC<br>TTGCTGTCGGGGACGGGCTCCTGGGCTTCACTGTC<br>CGGGACTCGGAGTCGACGAGGTTACTTTGAGTAAAT<br>TAGAGTGTTCAAAGCAGGCCTACGCTCTGAATACAT<br>TAGCATGGAATAACACGATAGGACTCTGGCCTATCT<br>TGTTGGTCTGTAGGACCGGAGTAATGATTAAGAGGG<br>ACAGTCGGGGGCATTCGTATTTCATTGTCAGAG	KM870706.1	Uncultured eukaryote clone TE203C 18S ribosomal RNA gene, complete sequence	100%	3e-165	100%
				AF514410.2	Raphidonema sempervirens strain CCCryo 011a-99 18S ribosomal RNA gene, partial sequence	100%	1e-163	99%

	TTTTACTT	18,169	CCAGCTCCAATAGCGTATATTTAAGTTGCTGCAGTTAAAAAGCTCGTAGTTGGATTTCGGATGGGTTCCGTCGGTCCGCCGTTTCGGTGTGCACTGACGGCGCCTATCTTGCTGTCGGGGACGGGCTCCTGGGCTTCACTGTCCGGGACTCGGAGTCGACGAGGTTACTTTGAGTAAATTAGAGTGTTCAAAGCAGGCCTACGCTCTGAATACATTAGCATGGAATAACACGATAGGACTCTGGCCTATCTTGTTGGTCTGTAGGACCGGAGTAATGATTAAGAGGGACAGTCGGGGGCATTCGTATTTCATTGTCAGAG	AF514410.2	Raphidonema sempervirens strain CCCryo 011a-99 18S ribosomal RNA gene, partial sequence	100%	3e-165	100%
				KM870742.1	Uncultured eukaryote clone TE208D 18S ribosomal RNA gene, complete sequence	100%	3e-165	100%
	TTTCCCTC	4,994	CCAGCTCCAATAGCGTATATTTAAGTTGCTGCAGTTAAAAAGCTCGTAGTTGGATTTCGGATGGGTTCCGTCGGTCCGCCGTTCCGGTGTGCACTGCCGGCGCCTATCTTGCTGTCGGGGACGGGCTCCTGGGCTTCACTGTCCGGGACCCGGAGTCGACGAGGTTACTTTGAGTAAATTAGAGTGTTCAAAGCAGGCCTACGCTCTGAATACATTAGCATGGAATAACACGATAGGACTCTGGCCTATCTTGTTGGTCTGTAGGACCGGAGTAATGATTAAGAGGGACAGTCGGGGGCATTCGTATTTCATTGTCAGAG	KM870651.1	Uncultured eukaryote clone TE107B 18S ribosomal RNA gene, complete sequence	100%	1e-163	99%
				KM870611.1	Raphidonema sempervirens strain CCCryo 011a-99 18S ribosomal RNA gene, partial sequence	100%	1e-163	99%
	TTCCACTT	941	CCAGCTCCAATAGCGTATATTTAAGTTGCTGCAGTTAAAAAGCTCGTAGTTGGATTTCGGATGGGTTCCGTCGGTCCGCCGTCCCGGTGTGCACTGACGGCGCCTATCTTGCTGTCGGGGACGGGCTCCTGGGCTTCACTGTCCGGGACTCGGAGTCGACGAGGTTACTTTGAGTAAATTAGAGTGTTCAAAGCAGGCCTACGCTCTGAATACATTAGCATGGAATAACACGATAGGACTCTGGCCTATCTTGTTGGTCTGTAGGACCGGAGTAATGATTAAGAGGGACAGTCGGGGGCATTCGTATTTCATTGTCAGAG	KM870650.1	Uncultured eukaryote clone TE107A 18S ribosomal RNA gene, complete sequence	100%	1e-163	99%
				AF514410.2	Raphidonema sempervirens strain CCCryo 011a-99 18S ribosomal RNA gene, partial sequence	100%	5e-162	99%
	GCACGCCT	70	CCAGCTCCAATAGCGTATATTTAAGTTGCTGCAGTTAAAAAGCTCGTAGTTGGATTTCGGATGGGTGCCGTCGGTCCGCCTCACGGTGTGTTACTGGCGGCGCCCATCTTGCTGTCGGGGACGGGCTCCTGGGCTTAACTGTCTGGGACTCGGAGTCGACGAGGTTACTTTGAGTAAATTAGAGTGTTCAAAGCAGGCCTACGCTCTGAATACATTAGCATGGAATAACACTATAGGACTCTGGCCTATCTTGTTGGTCTGTAGGACCGGAGTAATGATTAAGAGGGACAGTCGGGGGCATTCGTATTTCATTGTCAGAG	FJ490230.1	Uncultured Trebouxiophyceae clone H_27 18S ribosomal RNA gene, partial sequence	100%	3e-160	99%

**Uncultured Chlamydomonadaceae HW0117**	GGTATGC	30,640	CCAGCTCCAATAGCGTATATTTAAGTTGTTGCAGTTA<br>AAAAGCTCGTAGTTGGATTTCGGATGGGTATCAATG<br>GTCCGGTTCGCTGTGTACTGTTGCTGCCTATCTTTC<br>TGCCGGAAATGGGATCTTAGGCTTTACTGTCTGAGA<br>CCTAGAGTCGGCGAGGTTACTTTGAGTAAATTAGAG<br>TGTTCAAAGCAAGCTTACGCTCTGAATACATTAGCAT<br>GGAATAACACAATAGGACTTCGGCCTATCTTGTTGG<br>TCTGTAGGACTGAAGTAATGATTAAGAGGGACAGTC<br>GGGGGCATTCGTATTTCATTGTCAGAGGTG	GU117575.1	Uncultured Chlamydomonadaceae clone HW01/17 18S small subunit ribosomal RNA gene, partial sequence	100%	3e-160	99%
	GTAGTAG	810	CAGCTCCAATAGCGTATATTTAAGTTGTTGCAGTTAA<br>AAAGCTCGTAGTTGGATTTCGGATGGGTATCAATGG<br>TCCGGTTCGCTGTGTACTGTTGCTGCCTATCTTTCT<br>GCCGGAAATGGGATCTTAGGCTTTACTGTCTGAGAC<br>CTAGAGTCGGCGAGGTTACTTTGAGTAAATTAGAGT<br>GTTCAAAGCAAGCTTACGCTCTGAATACATTAGCAT<br>GGAATAACACAATAGGACTTCGGCCTATCTTGTTGG<br>TCTGTAGGACTGAAGTAATGATTAAGAGGGACAGTC<br>GGGGGCATTCGTATTTCATTGTCAGAGGTGA	GU117575.1	Uncultured Chlamydomonadaceae clone HW01/17 18S small subunit ribosomal RNA gene, partial sequence	100%	3e-160	99%
	GGTATTT	804	CCAGCTCCAATAGCGTATATTTAAGTTGTTGCAGTTA<br>AAAAGCTCGTAGTTGGATTTCGGATGGGTATCAATG<br>GTCCGGTTCGCTGTGTACTGTTGCTGCCTATCTTTC<br>TGCCGGAAATGGGATCTTAGGCTTTACTGTCTGAGA<br>CCTAGAGTCGGCGAGGTTACTTTGAGTAAATTAGAG<br>TGTTCAAAGCAAGCTTACGCTCTGAATACATTAGCAT<br>GGAATAACACAATAGGACTTCGGCCTATCTTGTTGG<br>TCTGTAGGACTGAAGTAATGATTAATAGGGACAGTT<br>GGGGGCATTCGTATTTCATTGTCAGAGGTG	GU117575.1	Uncultured Chlamydomonadaceae clone HW01/17 18S small subunit ribosomal RNA gene, partial sequence	100%	5e-157	98%
	GGTATAT	611	CCAGCTCCAATAGCGTATATTTAAGTTGTTGCAGTTA<br>AAAAGCTCGTAGTTGGATTTCGGATGGGTATCAATG<br>GTCCGGTTCGCTGTGTACTGTTGCTGCCTATCTTTC<br>TGCCGGAAATGGGATCTTAGGCTTTACTGTCTGAGA<br>CCTAGAGTCGGCGAGGTTACTTTGAGTAAATTAGAG<br>TGTTCAAAGCAAGCTTACGCTCTGAATACATTAGCAT<br>GGAATAACGTGATAGGACTCTGGTCCTATTGTGTTG<br>GTCTTCGGGACCGGAGTAATGATTAATAGGGACAGT<br>TGGGGGCATTCGTATTTCATTGTCAGAGGT	GU117575.1	Uncultured Chlamydomonadaceae clone HW01/17 18S small subunit ribosomal RNA gene, partial sequence	100%	9e-135	94%

	GGTATGT	584	CCAGCTCCAATAGCGTATATTTAAGTTGTTGCAGTTA<br>AAAAGCTCGTAGTTGGATTTCGGATGGGTATCAATG<br>GTCCGGTTCGCTGTGTACTGTTGCTGCCTATCTTTC<br>TGCCGGAAATGGGATCTTAGGCTTTACTGTCTGAGA<br>CCTAGAGTCGGCGAGGTTACTTTGAGTAAATTAGAG<br>TGTTCAAAGCAAGCTTACGCTCTGAATACATTAGCAT<br>GGAATAACACAATAGGACTTCGGCCTATCTTGTTGG<br>TCTGTAGGACTGAAGTAATGATTAAGAGGGACAGTT<br>GGGGGCATTCGTATTTCATTGTCAGAGGTG	GU117575.1	Uncultured Chlamydomonadaceae clone HW01/17 18S small subunit ribosomal RNA gene, partial sequence	100%	1e-158	99%
	TAAGCAT	506	CCAGCTCCAATAGCGTATATTTAAGTTGTTGCAGTTA<br>AAAAGCTCGTAGTTGGATTTCGGATGGGTATCAATG<br>GTCCGGTTCGCTGTGTACTGTTGCTGCCTATCTTTC<br>TGCCGGAAATGGGATCTTAGGCTTTACTGTCTGAGA<br>CCTAGAGTCGGCGATGTTACTTTGAAAAAATTAGAG<br>TGTTCAAAGCAGGCCTACGCTCTGAATACATTAGCA<br>TGGAATAACGTGATAGGACTCTGGTCCTATTGTGTT<br>GGTCTTCGGGACCGGAGTAATGATTAATAGGGACA<br>GTTGGGGGCATTCGTATTTCATTGTCAGAGGT	GU117575.1	Uncultured Chlamydomonadaceae clone HW01/17 18S small subunit ribosomal RNA gene, partial sequence	100%	2e-126	93%
	GGTGCAT	416	CCAGCTCCAATAGCGTATATTTAAGTTGTTGCAGTTA<br>AAAAGCTCGTAGTTGGATTTCGGATGGGTATCAATG<br>GTCCGGTTCGCTGTGTACTGTTGCTGCCTATCTTTC<br>TGCCGGAAATGGGATCTTAGGCTTTACTGTCTGAGA<br>CCTAGAGTCGGCGAGGTTACTTTGAGTAAATTAGAG<br>TGTTCAAAGCAGGCCTACGCTCTGAATACATTAGCA<br>TGGAATAACGTGATAGGACTCTGGTCCTATTGTGTT<br>GGTCTTCGGGACCGGAGTAATGATTAATAGGGACA<br>GTTGGGGGCATTCGTATTTCATTGTCAGAGGT	GU117575.1	Uncultured Chlamydomonadaceae clone HW01/17 18S small subunit ribosomal RNA gene, partial sequence	100%	2e-131	94%
	GAAGCAT	206	CCAGCTCCAATAGCGTATATTTAAGTTGTTGCAGTTA<br>AAAAGCTCGTAGTTGGATTTCGGATGGGTATCAATG<br>GTCCGGTTCGCTGTGTACTGTTGCTGCCTATCTTTC<br>TGCCGGAAATGGGATCTTAGGCTTTACTGTCTGAGA<br>CCTAGAGTCGGCGAGGTTACTTTGAAAAAATTAGAG<br>TGTTCAAAGCAGGCCTACGCTCTGAATACATTAGCA<br>TGGAATAACGTGATAGGACTCTGGTCCTATTGTGTT<br>GGTCTTCGGGACCGGAGTAATGATTAATAGGGACA<br>GTTGGGGGCATTCGTATTTCATTGTCAGAGGT	GU117575.1	Uncultured Chlamydomonadaceae clone HW01/17 18S small subunit ribosomal RNA gene, partial sequence	100%	4e-128	93%

**Uncultured Chlamydomonadaceae DR1WP27**	CTTCTGC	24,952	CCAGCTCCAATAGCGTATATTTAAGTTGTTGCAGTTA<br>AAAAGCTCGTAGTTGGATTTCGGGTGGGTTCGGTCG<br>GTCCGCCTCTGGTGTGCACTGATTGAGCCTTCCTTT<br>CTGCCGGGGACGGGTTCCTGGGCCTCACGGTTTGG<br>GACTCGGAGTCGGCGAGGTTACTTTGAGTAAATTAG<br>AGTGTTCAAAGCAAGCCTACGCTCTGAATACATTAG<br>CATGGAATAACGCGATAGGACTCTGGCCTATCTTGT<br>TGGTCTGTAGGACTGGAGTAATGATTAAGAGGGACA<br>GTCGGGGGCATTCGTATTTCATTGTCAGAGGT	KT184442.1	Uncultured eukaryote clone ALA5117773_P02_A09 18S ribosomal RNA gene, partial sequence	100%	3e-165	100%
				KM870766.1	Chlamydomonas nivalis strain CCCryo RS_0015-2010 18S ribosomal RNA gene, partial sequence	100%	3e-165	100%
	CTTCCAT	1,853	CCAGCTCCAATAGCGTATATTTAAGTTGTTGCAGTTA<br>AAAAGCTCGTAGTTGGATTTCGGGTGGGTTCGGTCG<br>GTCCGCCTCTGGTGTGCACTGATTGAGCCTTCCTTT<br>CTGCCGGGGACGGGTTCCTGGGCCTCACGGTTTGG<br>GACTCGGAGTCGGCGAGGTTACTTTGAGTAAATTAG<br>AGTGTTCAAAGCAAGCCTACGCTCTGAATACATTAG<br>CATGGAATAACGTGATAGGACTCTGGTCCTATTGTG<br>TTGGTCTTCGGGACCGGAGTAATGATTAATAGGGAC<br>AGTTGGGGGCATTCGTATTTCATTGTCAGAGG	KT184442.1	Uncultured eukaryote clone ALA5117773_P02_A09 18S ribosomal RNA gene, partial sequence	100%	4e-148	97%
				JQ790560.1	Chlamydomonas nivalis strain CCCryo RS_0015-2010 18S ribosomal RNA gene, partial sequence	100%	4e-148	97%
	TTACCGC	1,576	CCAGCTCCAATAGCGTATATTTAAGTTGTTGCAGTTA<br>AAAAGCTCGTAGTTGGATTTCGGGTGGGTTTGGTCG<br>GTCCGCCTCTGGTGTGCACTGACTGAGCGTACCTTT<br>CTGCCGGGGACGGGTTCCTGGGCCTCACGGTCTGG<br>GACTCGGAGTCGGCGAGGTTACTTTGAGTAATTTAG<br>AGTGTTCAAAGCAAGCCTACGCTCTGAATACATTAG<br>CATGGAATAACGCGATAGGACTCTGGCCTATCTTGT<br>TGGTCTGTAGGACCGGAGTAATGATTAAGAGGGACA<br>GTCGGGGGCATTCGTATTTCATTGTCAGAGGT	GU117586.1	Ploeotila sp. CCCryo086-99 strain CCCryo 086a-99 18S small subunit ribosomal RNA gene, partial sequence	100%	3e-160	99%
				KM870694.1	Uncultured eukaryote clone TE201E 18S ribosomal RNA gene, complete sequence	100%	5e-157	98%
				JQ790560.1	Chlamydomonas nivalis strain CCCryo RS_0015-2010 18S ribosomal RNA gene, partial sequence	100%	2e-156	98%
	TAAATCGC	977	CCAGCTCCAATAGCGTATATTTAAGTTGTTGCAGTTA<br>AAAAGCTCGTAGTTGGATTTCGGGTGGGTTTGGTCG<br>GTCCGCCTCTGGTGAGCACTGACTGAGCGTACCTTT<br>CTGCCGGGGACGGGTTCCTGGGCCTTACGGTCTGG<br>GACTCGGAGTCGGCGAGGTTACTTTGAGTAATTTAG<br>AGTGTTCAAAGCAAGCCTACGCTCTGAATACATTAG<br>CATGGAATAACGCGATAGGACTCTGGCCTATCTTGT<br>TGGTCTGTAGGACCGGAGTAATGATTAAGAGGGACA<br>GTCGGGGGCATTCGTATTTCATTGTCAGAGGT	GU117586.1	Ploeotila sp. CCCryo086-99 strain CCCryo 086a-99 18S small subunit ribosomal RNA gene, partial sequence	100%	5e-157	98%
				KM870694.1	Uncultured eukaryote clone TE201E 18S ribosomal RNA gene, complete sequence	100%	1e-153	98%
				JQ790560.1	Chlamydomonas nivalis strain CCCryo RS_0015-2010 18S ribosomal RNA gene, partial sequence	100%	3e-150	97%

CTTCTTT	583	CCAGCTCCAATAGCGTATATTTAAGTTGTTGCAGTTA<br>AAAAGCTCGTAGTTGGATTTCGGGTGGGTTCGGTCG<br>GTCCGCCTCTGGTGTGCACTGATTGAGCCTTCCTTT<br>CTGCCGGGGACGGGTTCCTGGGCCTCACGGTTTGG<br>GACTCGGAGTCGGCGAGGTTACTTTGAGTAAATTAG<br>AGTGTTCAAAGCAAGCCTACGCTCTGAATACATTAG<br>CATGGAATAACGCGATAGGACTCTGGCCTATCTTGT<br>TGGTCTGTAGGACTGGAGTAATGATTAATAGGGACA<br>GTTGGGGGCATTCGTATTTCATTGTCAGAGGT	KT184442.1	Uncultured eukaryote clone ALA5117773_P02_A09 18S ribosomal RNA gene, partial sequence	100%	5e-162	99%
			JQ790560.1	Chlamydomonas nivalis strain CCCryo RS_0015-2010 18S ribosomal RNA gene, partial sequence	100%	5e-162	99%
CTACTGC	491	CCAGCTCCAATAGCGTATATTTAAGTTGTTGCAGTTA<br>AAAAGCTCGTAGTTGGATTTCGGGTGGGCTCGGTC<br>GGTCCGCCTCTGGTGTGCACTGATTGAGCCTATCTT<br>TCTGCCGGGGACGGGTTCCTGGGCCTCACGGTTTG<br>GGACTCGGAGTCGGCGAGGTTACTTTGAGTAAATTA<br>GAGTGTTCAAAGCAAGCCTACGCTCTGAATACATTA<br>GCATGGAATAACGCGATAGGACTCTGGCCTATCTTG<br>TTGGTCTGTAGGACTGGAGTAATGATTAAGAGGGAC<br>AGTCGGGGGCATTCGTATTTCATTGTCAGAGGT	KT184442.1	Uncultured eukaryote clone ALA5117773_P02_A09 18S ribosomal RNA gene, partial sequence	100%	3e-160	99%
			JQ790560.1	Chlamydomonas nivalis strain CCCryo RS_0015-2010 18S ribosomal RNA gene, partial sequence	100%	3e-160	99%
			JQ790560.1	Chlamydomonas nivalis strain CCCryo RS_0015-2010 18S ribosomal RNA gene, partial sequence	100%	3e-165	100%
CTTCTGT	440	CCAGCTCCAATAGCGTATATTTAAGTTGTTGCAGTTA<br>AAAAGCTCGTAGTTGGATTTCGGGTGGGTTCGGTCG<br>GTCCGCCTCTGGTGTGCACTGATTGAGCCTTCCTTT<br>CTGCCGGGGACGGGTTCCTGGGCCTCACGGTTTGG<br>GACTCGGAGTCGGCGAGGTTACTTTGAGTAAATTAG<br>AGTGTTCAAAGCAAGCCTACGCTCTGAATACATTAG<br>CATGGAATAACGCGATAGGACTCTGGCCTATCTTGT<br>TGGTCTGTAGGACTGGAGTAATGATTAAGAGGGACA<br>GTTGGGGGCATTCGTATTTCATTGTCAGAGGT	KT184442.1	Uncultured eukaryote clone ALA5117773_P02_A09 18S ribosomal RNA gene, partial sequence	100%	1e-163	99%
			JQ790560.1	Chlamydomonas nivalis strain CCCryo RS_0015-2010 18S ribosomal RNA gene, partial sequence	100%	1e-163	99%
TTTCTGC	427	CCAGCTCCAATAGCGTATATTTAAGTTGTTGCAGTTA<br>AAAAGCTCGTAGTTGGATTTTGGGTTGGTGTGGTCG<br>GTCCGCCTCTGGTGTGCACTGATTGAGCCTTCCTTT<br>CTGCCGGGGACGGGTTCCTGGGCCTCACGGTTTGG<br>GACTCGGAGTCGGCGAGGTTACTTTGAGTAAATTAG<br>AGTGTTCAAAGCAAGCCTACGCTCTGAATACATTAG<br>CATGGAATAACGCGATAGGACTCTGGCCTATCTTGT<br>TGGTCTGTAGGACTGGAGTAATGATTAAGAGGGACA<br>GTCGGGGGCATTCGTATTTCATTGTCAGAGGT	KT184442.1	Uncultured eukaryote clone ALA5117773_P02_A09 18S ribosomal RNA gene, partial sequence	100%	1e-158	99%
			JQ790560.1	Chlamydomonas nivalis strain CCCryo RS_0015-2010 18S ribosomal RNA gene, partial sequence	100%	1e-158	99%

CTTCCTT	252	CCAGCTCCAATAGCGTATATTTAAGTTGTTGCAGTTA<br>AAAAGCTCGTAGTTGGATTTCGGGTGGGTTCGGTCG<br>GTCCGCCTCTGGTGTGCACTGATTGAGCCTTCCTTT<br>CTGCCGGGGACGGGTTCCTGGGCCTCACGGTTTGG<br>GACTCGGAGTCGGCGAGGTTACTTTGAGTAAATTAG<br>AGTGTTCAAAGCAAGCCTACGCTCTGAATACATTAG<br>CATGGAATAACGCGATAGGACTCTGGCCTATCTTGT<br>TGGTCTGTAGGACCGGAGTAATGATTAATAGGGACA<br>GTTGGGGGCATTCGTATTTCATTGTCAGAGGT	KT184442.1	Uncultured eukaryote clone ALA5117773_P02_A09 18S ribosomal RNA gene, partial sequence	100%	3e-160	99%
			JQ790560.1	Chlamydomonas nivalis strain CCCryo RS_0015-2010 18S ribosomal RNA gene, partial sequence	100%	3e-160	99%
CTTATGC	103	CCAGCTCCAATAGCGTATATTTAAGTTGTTGCAGTTA<br>AAAAGCTCGTAGTTGGATTTCGGGTGGGTTCGGTCG<br>GTCCGCCTCTGGTGTGCACTGATTGAGCCTTCCTTT<br>CTGCCGGGGACGGGTTCCTGGGCCTAACGGTTTGG<br>GACTCGGAGTCGGCGAGGTTACTTTGAGTAAATTAG<br>AGTGTTCAAAGCAAGCCTACGCTCTGAATACATTAG<br>CATGGAATAACGCGATAGGACTCTGGCCTATCTTGT<br>TGGTCTGTAGGACTGGAGTAATGATTAAGAGGGACA<br>GTCGGGGGCATTCGTATTTCATTGTCAGAGGT	KM870746.1	Uncultured eukaryote clone TE208H 18S ribosomal RNA gene, complete sequence	100%	3e-165	100%
			JQ790560.1	Chlamydomonas nivalis strain CCCryo RS_0015-2010 18S ribosomal RNA gene, partial sequence	100%	1e-163	99%
CTTCCGC	53	CCAGCTCCAATAGCGTATATTTAAGTTGTTGCAGTTA<br>AAAAGCTCGTAGTTGGATTTCGGGTGGGTTCGGTCG<br>GTCCGCCTCTGGTGTGCACTGATTGAGCCTTCCTTT<br>CTGCCGGGGACGGGTTCCTGGGCCTCACGGTTTGG<br>GACTCGGAGTCGGCGAGGTTACTTTGAGTAAATTAG<br>AGTGTTCAAAGCAAGCCTACGCTCTGAATACATTAG<br>CATGGAATAACGCGATAGGACTCTGGCCTATCTTGT<br>TGGTCTGTAGGACCGGAGTAATGATTAAGAGGGACA<br>GTCGGGGGCATTCGTATTTCATTGTCAGAGGT	KT184442.1	Uncultured eukaryote clone ALA5117773_P02_A09 18S ribosomal RNA gene, partial sequence	100%	1e-163	99%
			JQ790560.1	Chlamydomonas nivalis strain CCCryo RS_0015-2010 18S ribosomal RNA gene, partial sequence	100%	1e-163	99%
TTACCAT	35	CCAGCTCCAATAGCGTATATTTAAGTTGTTGCAGTTA<br>AAAAGCTCGTAGTTGGATTTCGGGTGGGTTTGGTCG<br>GTCCGCCTCTGGTGTGCACTGACTGAGCGTACCTTT<br>CTGCCGGGGACGGGTTCCTGGGCCTCACGGTCTGG<br>GACTCGGAGTCGGCGAGGTTACTTTGAGTAATTTAG<br>AGTGTTCAAAGCAAGCCTACGCTCTGAATACATTAG<br>CATGGAATAACGTGATAGGACTCTGGTCCTATTGTG<br>TTGGTCTTCGGGACCGGAGTAATGATTAATAGGGAC<br>AGTTGGGGGCATTCGTATTTCATTGTCAGAGG	GU117586.1	Ploeotila sp. CCCryo086-99 strain CCCryo 086a-99 18S small subunit ribosomal RNA gene, partial sequence	100%	9e-145	96%
			KM870694.1	Uncultured eukaryote clone TE201E 18S ribosomal RNA gene, complete sequence	100%	2e-141	96%
			JQ790560.1	Chlamydomonas nivalis strain CCCryo RS_0015-2010 18S ribosomal RNA gene, partial sequence	100%	4e-138	95%

TTACCGT	24	CCAGCTCCAATAGCGTATATTTAAGTTGTTGCAGTTAAAAAGCTCGTAGTTGGATTTCGGGTGGGTTTGGTCGGTCCGCCTCTGGTGTGCACTGACTGAGCGTACCTTTCTGCCGGGGACGGGTTCCTGGGCCTCACGGTCTGGGACTCGGAGTCGGCGAGGTTACTTTGAGTAATTTAGAGTGTTCAAAGCAAGCCTACGCTCTGAATACATTAGCATGGAATAACGCGATAGGACTCTGGCCTATCTTGTTGGTCTGTAGGACCGGAGTAATGATTAAGAGGGACAGTTGGGGGCATTCGTATTTCATTGTCAGAGGT	GU117586.1	Ploeotila sp. CCCryo086-99 strain CCCryo 086a-99 18S small subunit ribosomal RNA gene, partial sequence	100%	1e-158	99%
			KM870694.1	Uncultured eukaryote clone TE201E 18S ribosomal RNA gene, complete sequence	100%	3e-155	98%
			JQ790560.1	Chlamydomonas nivalis strain CCCryo RS_0015-2010 18S ribosomal RNA gene, partial sequence	100%	6e-152	98%
TTACCTT	17	CCAGCTCCAATAGCGTATATTTAAGTTGTTGCAGTTAAAAAGCTCGTAGTTGGATTTCGGGTGGGTTTGGTCGGTCCGCCTCTGGTGTGCACTGACTGAGCGTACCTTTCTGCCGGGGACGGGTTCCTGGGCCTCACGGTCTGGGACTCGGAGTCGGCGAGGTTACTTTGAGTAATTTAGAGTGTTCAAAGCAAGCCTACGCTCTGAATACATTAGCATGGAATAACGCGATAGGACTCTGGCCTATCTTGTTGGTCTGTAGGACCGGAGTAATGATTAATAGGGACAGTTGGGGGCATTCGTATTTCATTGTCAGAGGT	GU117586.1	Ploeotila sp. CCCryo086-99 strain CCCryo 086a-99 18S small subunit ribosomal RNA gene, partial sequence	100%	5e -157	98%
			KM870694.1	Uncultured eukaryote clone TE201E 18S ribosomal RNA gene, complete sequence	100%	1e-153	98%
			JQ790560.1	Chlamydomonas nivalis strain CCCryo RS_0015-2010 18S ribosomal RNA gene, partial sequence	100%	3e-150	97%

Supplementary Figure 1: The oligotype alignments for each taxon, with oligotype names being derived from the nucleotide variations in the sequence alignment.

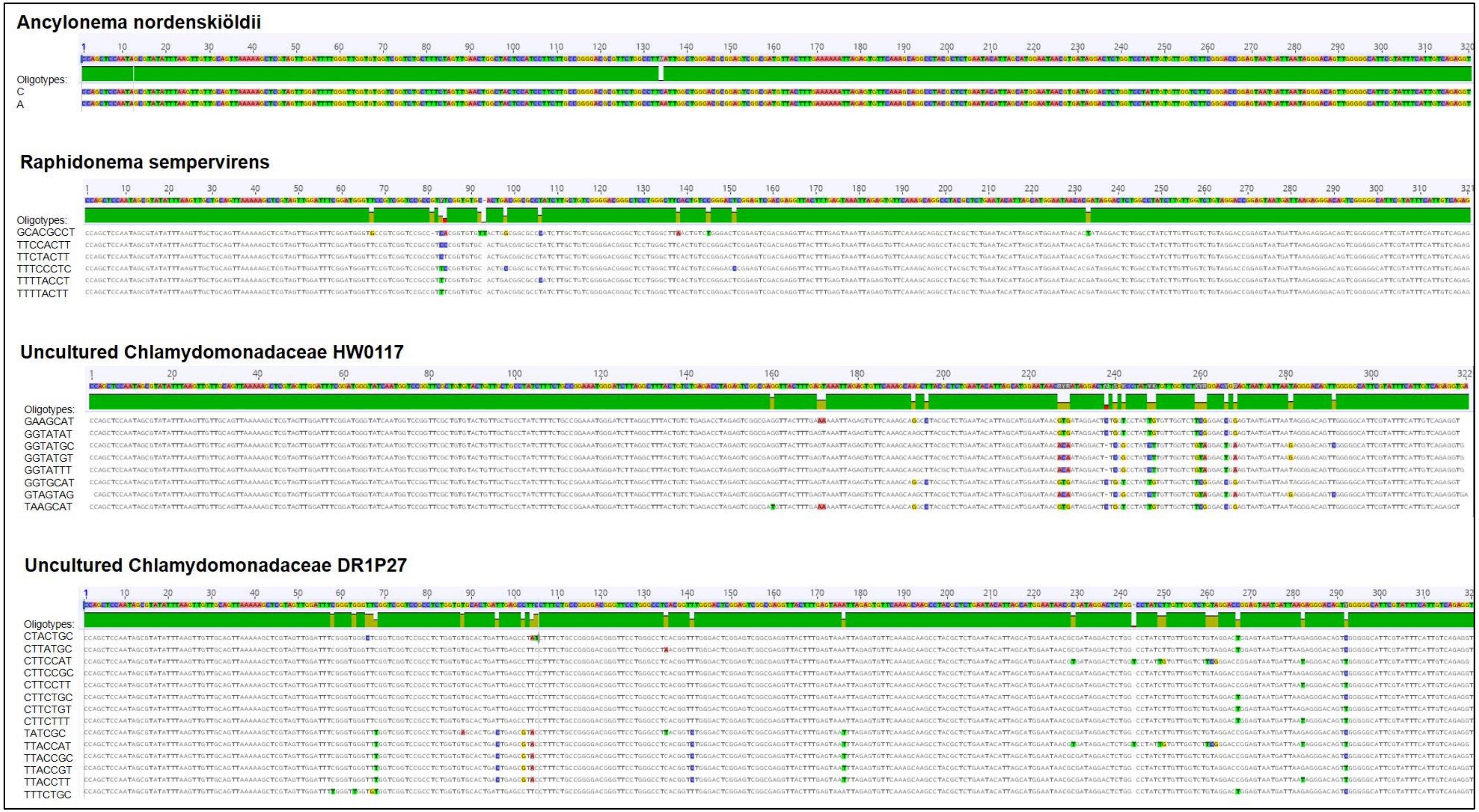

Supplementary Figure 2: Daily averaged temperature data for the period between the 1st of May and the 31st of August 2016 have been obtained from 3 weather stations, which are operating in close proximity to the sampling sites. Data for KAN_M (3 km away from site 3) and KAN_U (at site 1a) were provided by the Geological Survey of Denmark and Greenland (GEUS) (53) and data from S6 (2 km away from our base camp) were provided by the by the Institute for Marine and Atmospheric Research (IMAU) at Utrecht University. Graph shows a progressive decrease in average temperatures from the base camp (S6) to further inland (KAN_U). Those data can be used as a proxy for the extent of melting.

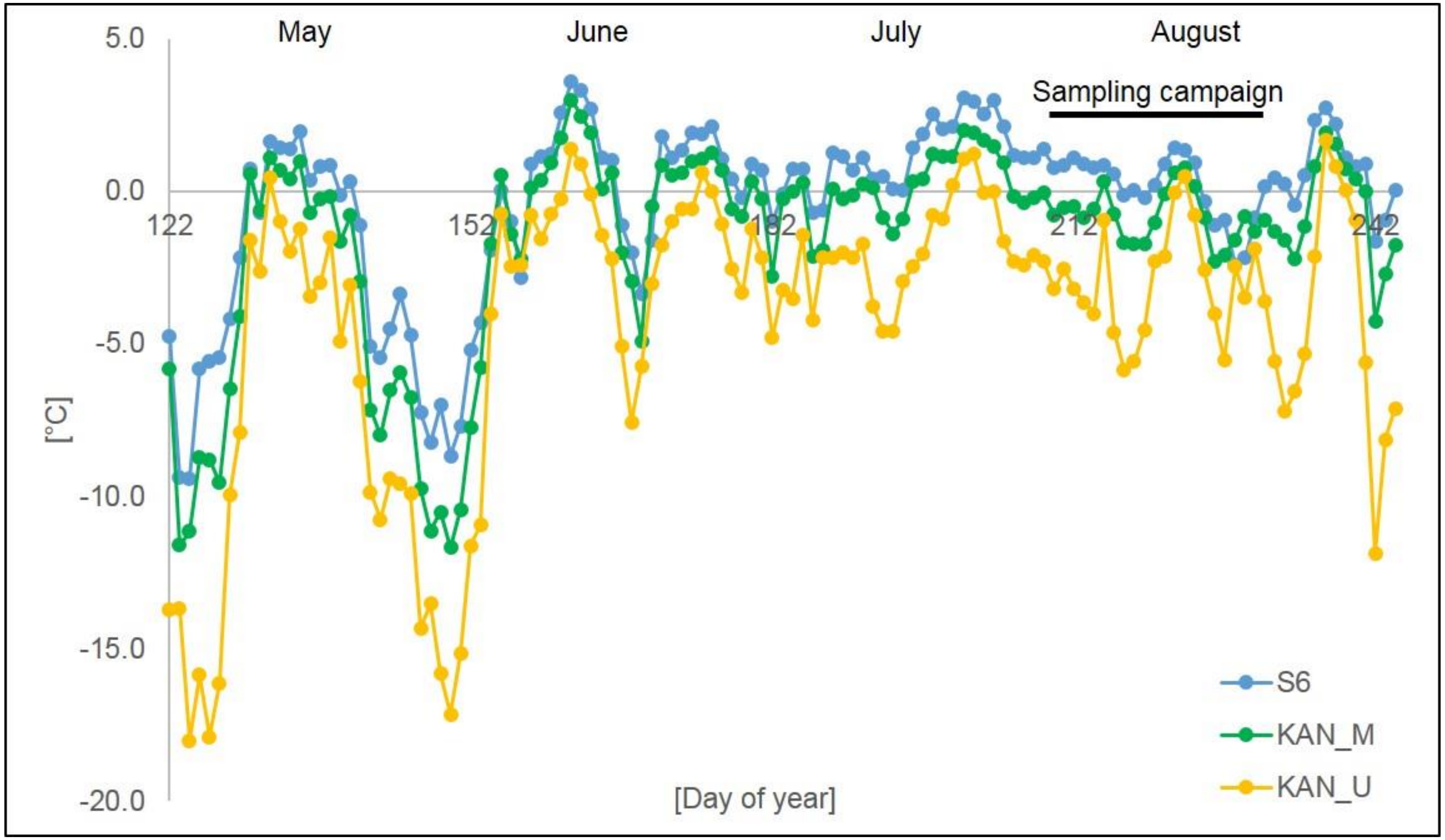

Supplement: Supplementary File 1 [file mgen-4-159-s001.pdf]
